# Differences in olfactory bulb mitral cell spiking with ortho- and retronasal stimulation revealed by data-driven models

**DOI:** 10.1371/journal.pcbi.1009169

**Published:** 2021-09-20

**Authors:** Michelle F. Craft, Andrea K. Barreiro, Shree Hari Gautam, Woodrow L. Shew, Cheng Ly

**Affiliations:** 1 Department of Statistical Sciences and Operations Research, Virginia Commonwealth University, Richmond, Virginia, United States of America; 2 Department of Mathematics, Southern Methodist University, Dallas, Texas, United States of America; 3 Department of Physics, University of Arkansas, Fayetteville, Arkansas, United States of America; National Research Council, ITALY

## Abstract

The majority of olfaction studies focus on orthonasal stimulation where odors enter via the front nasal cavity, while retronasal olfaction, where odors enter the rear of the nasal cavity during feeding, is understudied. The coding of retronasal odors via coordinated spiking of neurons in the olfactory bulb (**OB**) is largely unknown despite evidence that higher level processing is different than orthonasal. To this end, we use multi-electrode array *in vivo* recordings of rat OB mitral cells (**MC**) in response to a food odor with both modes of stimulation, and find significant differences in evoked firing rates and spike count covariances (i.e., noise correlations). Differences in spiking activity often have implications for sensory coding, thus we develop a single-compartment biophysical OB model that is able to reproduce key properties of important OB cell types. Prior experiments in olfactory receptor neurons (**ORN**) showed retro stimulation yields slower and spatially smaller ORN inputs than with ortho, yet whether this is consequential for OB activity remains unknown. Indeed with these specifications for ORN inputs, our OB model captures the salient trends in our OB data. We also analyze how first and second order ORN input statistics dynamically transfer to MC spiking statistics with a phenomenological linear-nonlinear filter model, and find that retro inputs result in larger linear filters than ortho inputs. Finally, our models show that the temporal profile of ORN is crucial for capturing our data and is thus a distinguishing feature between ortho and retro stimulation, even at the OB. Using data-driven modeling, we detail how ORN inputs result in differences in OB dynamics and MC spiking statistics. These differences may ultimately shape how ortho and retro odors are coded.

## Author summary

Olfaction is a key sense for many cognitive and behavioral tasks, and is particularly unique because odors can naturally enter the nasal cavity from the front or rear, i.e., ortho- and retro-nasal, respectively. Yet little is known about the differences in coordinated spiking in the olfactory bulb with ortho versus retro stimulation, let alone how these different modes of olfaction may alter coding of odors. We simultaneously record many cells in rat olfactory bulb to assess the differences in spiking statistics, and develop a biophysical olfactory bulb network model to study the reasons for these differences. Using theoretical and computational methods, we find that the olfactory bulb transfers input statistics differently for retro stimulation relative to ortho stimulation. Furthermore, our models show that the temporal profile of inputs is crucial for capturing our data and is thus a distinguishing feature between ortho and retro stimulation, even at the olfactory bulb. Understanding the spiking dynamics of the olfactory bulb with both ortho and retro stimulation is a key step for ultimately understanding how the brain codes odors with different modes of olfaction.

## Introduction

Olfactory processing naturally occurs in two distinct modes: orthonasal (**ortho**) where odors enter the front of the nasal cavity and retronasal (**retro**) where odors enter the rear through the throat. Orthonasal olfaction is essential for avoiding predators [1, 2], social interactions, and finding food, and has been studied most extensively in olfaction research. Retronasal olfaction is far less studied, but has a critical role in eating behaviors as chewed foods generate odorants that enter the nasal cavity upon exhalation. Retronasal olfaction drives flavor perception [3–5] and aids in avoiding harmful foods. Moreover, studies have shown that olfactory dysfunction with food odors is directly linked to obesity [6–8]. Previous studies have reported differences in cortical fMRI BOLD signals for ortho versus retro stimuli [9] and recent evidence suggests that food odors are easier to recognize when delivered retronasally versus orthonasally [10]. Calcium imaging studies have shown that the input to olfactory bulb from the nose differs for ortho versus retro stimulation [11]. However, the neural mechanisms that differentiate ortho versus retro olfactory processing at the level of spiking activity in olfactory bulb remain unknown.

**Fig 1:**
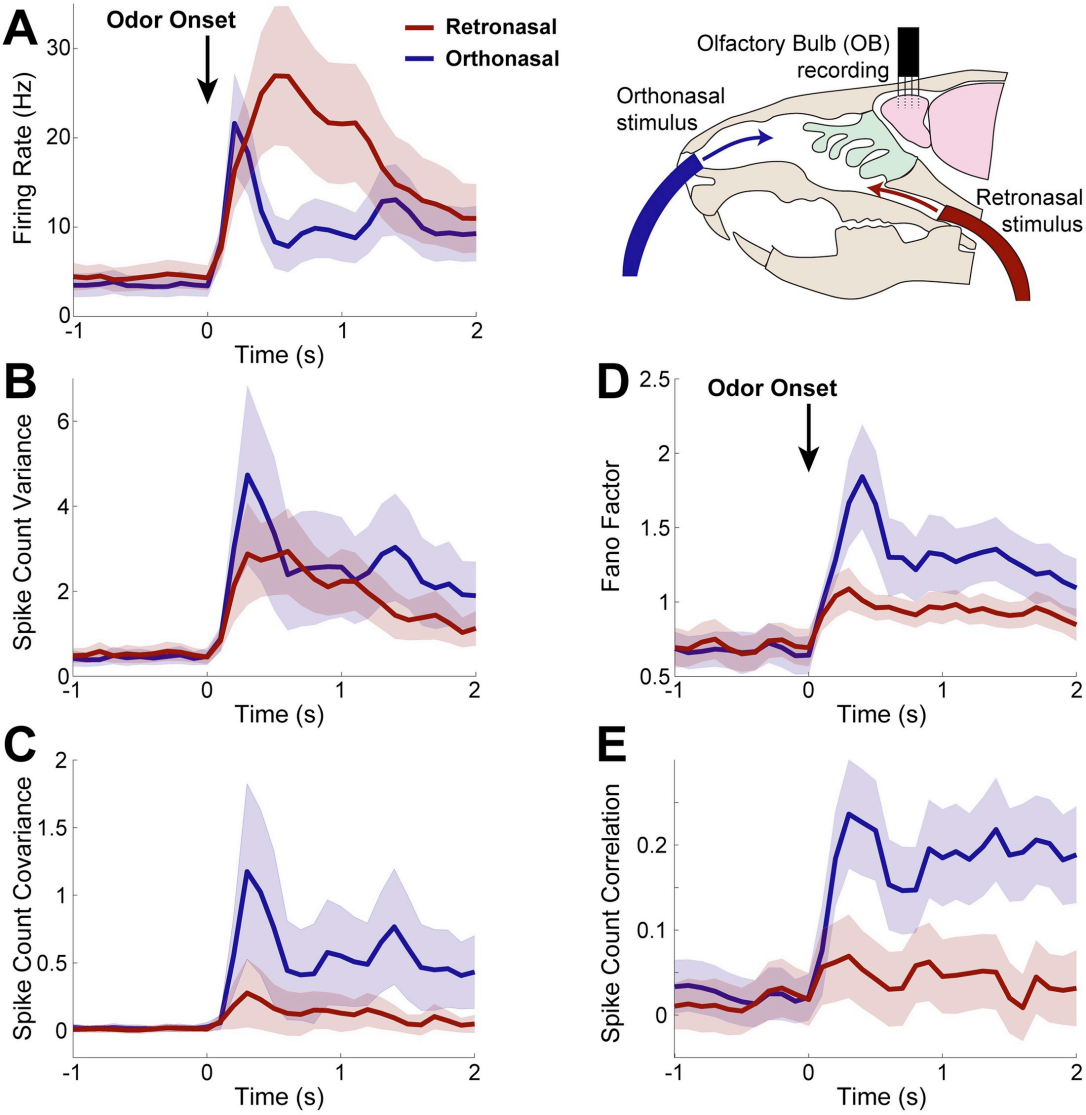
Spike statistics from *in vivo* multi-electrode array recordings. Population average spike statistics for orthonasal (blue) and retronasal (red) with stimulus onset at time *t* = 0 s as indicated by black arrow for 1 s duration. **A)** Firing rate (Hz) is statistically significantly different between ortho and retro for the duration of the evoked period (0.4 ≤ *t* ≤ 1.1 s). **B)** Spike count variance has no statistically significant difference between ortho and retro. **C)** Covariance of spike counts are statistically significant different throughout the evoked state (0 ≤ *t* ≤ 2) with ortho having larger values. Scaled measures of variability shown for completeness: Fano Factor **(D)** is the variance divided by mean spike count, and Pearson’s correlation **(E)** is the covariance divided by the product of the standard deviations; both are also different with ortho versus retro. Spike counts in 100 ms half-overlapping time windows averaging over all 10 trials. Significance: two-sample t-tests (assuming unequal variances) for each time bin to assess differences in population means, *p* < 0.01, also see S1 and S2 Figs. From 94 total cells and 1435 simultaneously recorded cell pairs; shaded regions show relative population heterogeneity: *μ* ± 0.2std (standard deviation across the population/pairs after trial-averaging; 0.2 scale for visualization).

Odor information is primarily processed in the olfactory bulb (**OB**) and then subsequently relayed to cortical areas via mitral cell (**MC**) (and tufted cell) spiking. Thus, any differences in MC spiking between ortho and retro are related to both the efficiency and accuracy [12–15] of odor coding, but any such differences are largely unknown. Presynaptic to the OB are olfactory receptor neurons (**ORN**s) whose activity is known to differ for ortho versus retro stimulation, as observed in prior imaging studies with fMRI [16], calcium imaging [11], and optical imaging in transgenic mice [17]. These and other prior studies [18–20] suggest that ORN synaptic inputs is a key factor for differences in OB activity. The two routes of stimulation make contact on different locations of the olfactory epithelium (shown in light green in Fig 1 experimental diagram) and thus activate different ORN receptor types within the epithelium. However, the implications of these differences in ORN activity for MC spiking have yet to be explored.

We perform *in vivo* recordings of rat OB mitral cells using multi-electrode arrays with a food odor (Ethyl Butyrate) stimulus, delivered by both modes of stimulation, to determine whether differences exist. We find significant differences in odor-evoked MC spiking with ortho versus retro stimulation in both firing rate (larger with retro) and spike count covariance (larger with ortho). However, understanding how retro stimulation can elicit both larger firing rates and smaller co-variablity than ortho is generally difficult in recurrent networks because of the numerous attributes that shape spike statistics [21–24]. Additionally, dissecting how components of ORN inputs alter OB spiking is difficult experimentally due to the complexity of both the recurrent circuitry in the OB [25, 26] and resulting spatiotemporal ORN responses [18, 20]. So we develop a single-compartment biophysical OB model that accounts for differences in ORN input to investigate how they affect MC spiking responses. Specifically, we model ORN input as a time-varying inhomogeneous Poisson Process [27], where the input rate has slower increase and decay for retro than ortho [11, 17], and the ORN input correlation is smaller for retro than ortho [11, 17]. With these specifications, our biophysical OB network model is able to capture the salient ortho versus retro MC spiking response trends in our experimental data.

However, our biophysical OB model is too complex to directly analyze mathematically in order to address the neural encoding problem of characterizing how MCs convert ORN input to spike responses. We use a simple linear-nonlinear (**LN**) model framework to assess how our biophysical OB network transfers input statistics (from ORN) to outputs (MC spike statistics). We find that the linear filter component of the LN model, i.e., convolution with ORN inputs, consistently has larger absolute values with retro than with ortho input. Thus the OB network model is more sensitive to ORN fluctuations with retro-like inputs than with ortho. Finally, we use our models to examine which key attribute(s) of ORN inputs (temporal profile, amplitude, input correlation) are most significant for capturing our data. We find that temporal profile is the critical attribute for ortho versus retronasal stimulus response.

This work provides a framework for how to analyze the sources driving different OB spiking responses to different modes of olfaction, as well as important insights that have implications for how the brain codes odors.

## Results

We performed *in vivo* multi-electrode array recordings of the OB in the mitral cell layer of anesthetized rats (see Materials and methods: **Electrophysiological recordings**) to capture odor evoked spiking activity of populations of putative MCs. This yielded a large number of cells (94) and simultaneously recorded pairs of cells (1435) with which to assess population average spiking statistics. The spike statistics are trial-averaged responses of a rat to a single odorant, Ethyl Butyrate (food odor). We focus on a food odor because they dominate retronasal smells, and a recent study showed that humans can more accurately detect food odors (vs. non-food odors) delivered retronasally [10]. In addition, an fMRI study showed different cortical activity [9] in humans for ortho versus retronasal stimulus, specifically with food odors.

The first and second order spike statistics are summarized in Fig 1, including the firing rate (peri-stimulus time histogram, **PSTH**, Fig 1A), the spike count variance (Fig 1B), the spike count covariance (Fig 1C), Fano Factor (variance divided by mean, Fig 1D), and Pearson’s correlation (Fig 1E). For each cell and simultaneously record pair of cells, we calculated the trial-averaged spike statistics with half-overlapping 100 ms time windows. The time window 100 ms is an intermediate value between shorter (membrane time constants, AMPA, GABA_A_, etc.) and longer time scales (NMDA, calcium, and other ionic currents) known to exist in the OB.

We find statistically significant differences between ortho and retro stimulation in almost all of the first and second order MC spike count statistics. At odor onset, orthonasal stimulation elicits larger firing rates with a faster rise than retronasal, after which retronasal firing is larger and remains elevated longer than with orthonasal. These trends are consistent with imaging studies of the glomeruli layer in OB in transgenic mice (see [17], their Fig 2) as well as EOG recordings of the superficial layers of the OB in rats (see [19], their Fig 7). More specifically, we find statistical significance (*α* = 0.01) between ortho- and retronasal firing rate after and for the duration of the odor stimulation. We also find that MC spike count covariance for ortho is significantly larger than retro for the entirety of the evoked state. MC spike count variance, however, is not significantly different. Note that we specifically tested whether the population averages (averaged over all cells for PSTH and spike variance, over all simultaneously recorded pairs for spike covariance) are significantly different between ortho and retro, via a two-sample t-test assuming unequal variances with the null hypothesis of equal population averages (see S1 Fig). Further, we calculated Cohen’s *d* value to measure effect size [28], and find medium effect size for the majority of the evoked time period for all spike statistics considered except for variance, see S2 Fig.

Hereafter, we mainly focus on understanding the differences in firing rate and spike count covariance because they directly impact common coding metrics (e.g. the Fisher information) in contrast to scaled measures of variability (Fano factor and Pearson’s correlation) that are nonlinear functions of the entities that impact coding [12]. Moreover, Fano factor and correlation both depend on variance, which is not statistically different with ortho and retro (but see S1(D) and S1(E) Fig for completeness).

### OB network model captures data trends

**Fig 2:**
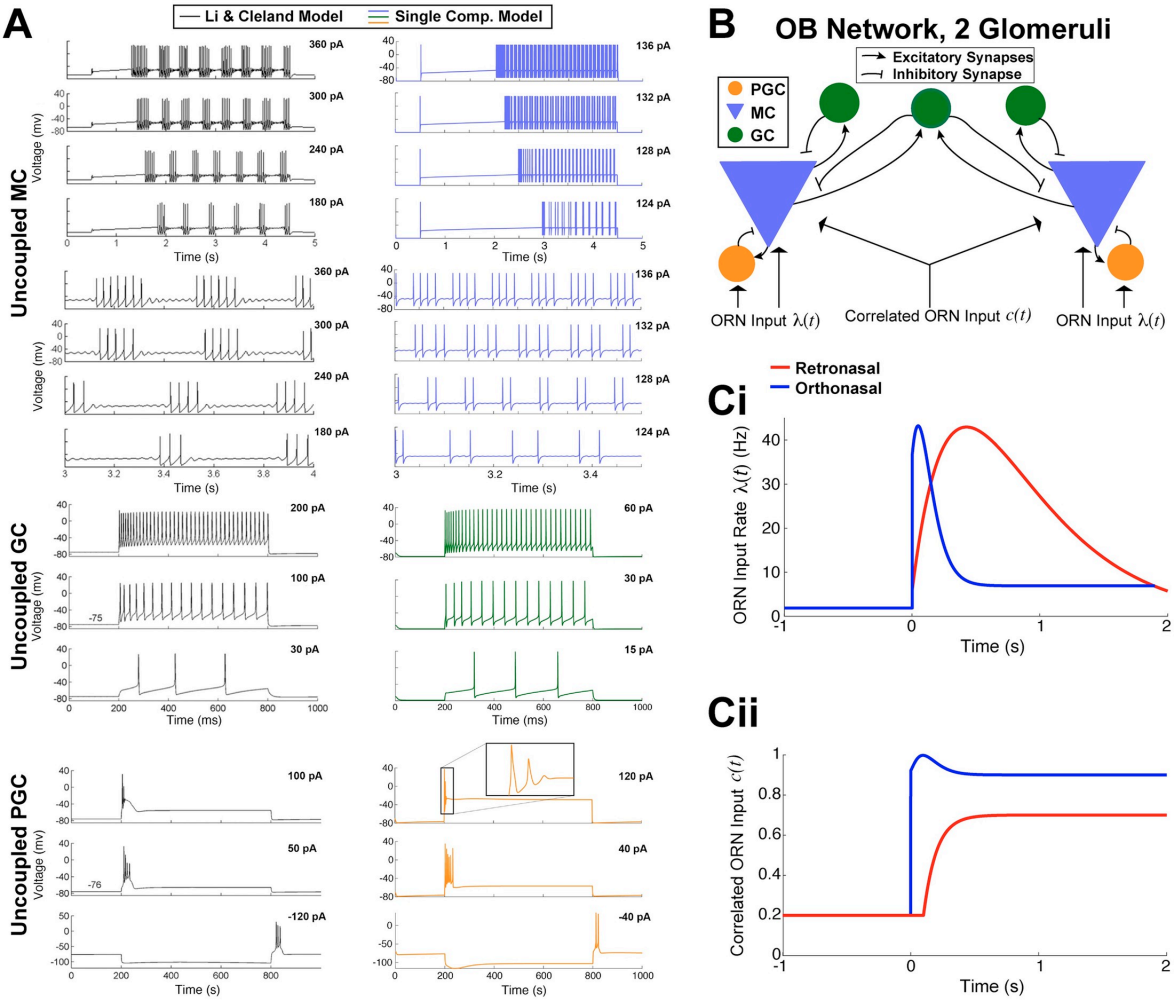
Biophysical OB model. **A)** Dynamics of the 3 uncoupled cell models. MC voltage dynamics with current step inputs in Li & Cleland models (black curves on the left, copied from Li & Cleland [29]) are captured by our single-compartment model (blue on the right). Rows 5–8 show expanded time view of first 4 rows to highlight spike cluster sizes and sub-threshold oscillations (same voltage axis for each). GC voltage responses to three different levels of current injection in the Li & Cleland model (black curves on the left) is similar to our model (green on the right). PGC responses with depolarizing current steps again are similar in both models. Note that release from a hyperpolarizing current injection leads to transient spiking in both models (bottom). **B)** Coupled OB network model of 2 glomeruli with ORN inputs. ORN synapses are driven by correlated inhomogeneous Poisson Processes (Eqs (10)–(12)). **C)** Based on ORN imaging studies, we set λ_*O*_(*t*) to increase and decay faster than λ_*R*_(*t*) with odor onset at time 0s (i). Similarly, we set the input correlation of ORN synapses to the 2 MCs to *c*_*R*/*O*_(*t*) where *c*_*R*_(*t*) < *c*_*O*_(*t*) and *c*_*O*_(*t*) rises quicker than *c*_*R*_(*t*) (ii).

To better understand how differences in MC spiking with ortho and retro stimulation come about, we developed a single-compartment OB network model based on Li & Cleland’s multi-compartment model [29, 30]. Our model is more computationally efficient than their larger multi-compartment models [29, 30], requiring a fraction of the variables (tens of state variables instead of thousands). Importantly, our single-compartment model retains important biophysical features (Fig 2A).

In Fig 2A, we see in both models of MC (uncoupled) that the time to spiking decreases with increasing current values, and the number of spikes in a cluster increases with current values consistent with prior electrophysiological experiments [31–33]. The spacing between spike clusters and number of spikes in a cluster in our model (right) qualitatively match the Li & Cleland model (left). The sub-threshold oscillations are not as prominent as in Li & Cleland, but still apparent. In the uncoupled GC models, both ours and Li & Cleland’s models exhibit a delay to the first spike with weak current step [34] (Fig 2A, bottom) and tonic firing without appreciable delay for higher current injections [35] (Fig 2A, middle and top). In the uncoupled PGC models, we do not observe repetitive firing in either models (Fig 2A, top and middle). Also, releasing from a hyperpolarizing current injection (bottom) can illicit spiking in both models, as observed by McQuiston & Katz [36]. Thus, we have a condensed OB model by using far less equations than Cleland’s models while retaining many of the biophysical dynamics known to exist in these 3 important OB cell types.

Since our focus is on first and second order population-averaged spiking statistics, we use a minimal OB network model with 2 glomeruli (Fig 2B). Each glomerulus has a PGC, MC and GC; we also include a common GC that provides shared inhibition to both MCs because GCs are known to span multiple glomeruli and shape MC spike correlation [26, 37, 38]. Within the OB network, the PGC and GC cells provide presynaptic GABA_A_ inhibition to the MCs they are coupled to, while MC provide both AMPA and NMDA excitation to PGC and GCs (see Materials and methods: **Single-Compartment Biophysical Model** for further details). The ORN synaptic inputs are an important component of this coupled OB network; they are driven by correlated inhomogeneous Poisson Process with increases in rate and correlation at odor onset. The specific time-varying input rate and correlation we use are shown in Fig 2Ci and 2Cii, respectively. The differences in ortho versus retro (Fig 2Ci and 2Cii) are based on prior studies of ORN input to the OB in response to both ortho and retro stimulation [11, 17]. We fixed all model components and manually varied the ORN input rate λ_*O*/*R*_(*t*), see Materials and methods: **Fitting biophysical network model to data** for further details.

**Fig 3:**
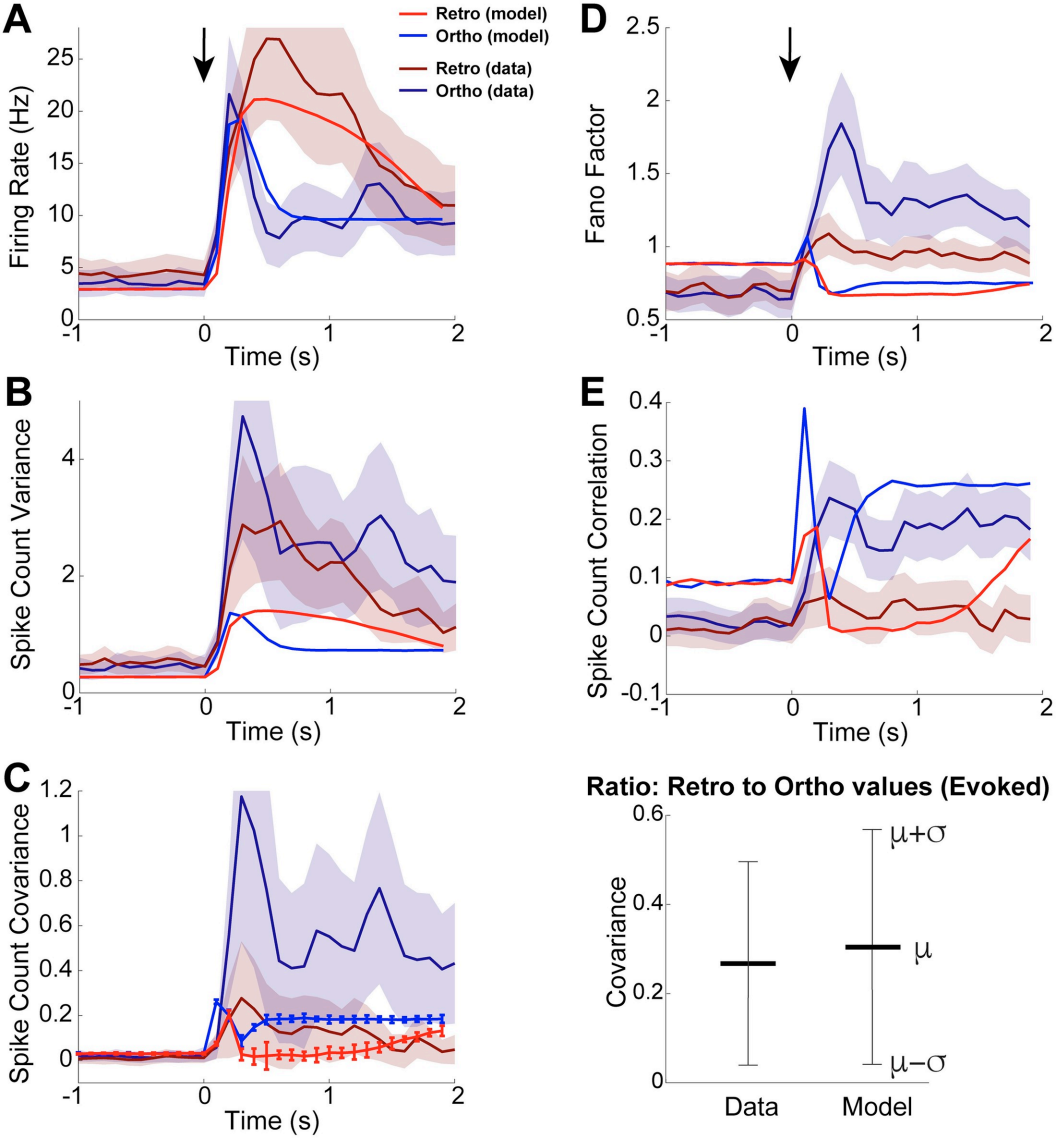
OB model captures trends in our data. Comparison of all first and second-order statistics of coupled OB network model to our data. **A)** Firing rate of ortho increases and decays faster than retro in both data and model. **B)** Variance of spike counts for ortho and retro shown for completeness, but recall that in experimental data that they are not statistically different. **C)** Covariance of spike counts is larger for ortho than retro in both data and model (left), but the magnitudes of data and model differ. Comparison of the ratio of retro covariance to ortho covariance in the evoked state (right) shows that the model captures the relative differences between ortho and retro—here *μ* (resp. *σ*) is the average (resp. standard deviation) ratio over 20 time bins in the evoked state. For **A–C**, top shaded error regions of data (retro in **A**, ortho in **B,C**) are cut-off to better compare model and data. Comparisons of the (**D**) Fano factor and (**E**) Pearson’s spike count correlation shown for completeness despite both measures depending on spike count variance. **D)** The model has slightly larger Fano factor with ortho, consistent with the data. **E)** The model does qualitatively capture the spike count correlation for both ortho and retro, at least in the evoked state.

A comparison of first and second-order statistics between our OB model and *in vivo* data is shown in Fig 3. With the ORN activity specified in Fig 2C, our OB model is able to qualitatively capture trends seen in our data for firing rate and spike count covariance. Firing rates in Fig 3A show that both the model and data exhibit larger firing rates for ortho at odor onset followed by a sharper decline. After the initial increase in ortho firing rates, retro firing rates continue to increase, eventually becoming larger than ortho and remaining elevated longer, consistent with optical imaging experiments (see [17] their Fig 2). Although there is no significant difference in the spike count variance between ortho and retro in our experimental data, we show our data with model for completeness (Fig 3B).

Our OB model captures the trend that ortho spike count covariance is larger than retro after odor onset, Fig 3C (left). The OB model certainly does not capture the magnitude of the spike count covariance in the data; recall that covariance in our experimental data is the population average over all 1435 simultaneously recorded pairs with significant heterogeneity while our model is homogeneous. But the relative differences between retro and ortho (as measured by the ratio of retro to ortho covariance in the evoked state) are similar (Fig 3C, right). Thus our OB model captures the salient trends of the population-averaged spike count statistics. We also show comparisons of Fano Factor (Fig 3D) and Pearson’s correlation (Fig 3E) for completeness. Consistent with our data, our OB model has larger Fano Factor and spike count correlation for ortho than with retro. In the evoked state, the OB model matches spike count correlation for both ortho and retro well. The larger ortho Fano factor in our data is captured in our model, but the difference is very modest.

### How OB network transfers ORN input statistics

**Fig 4:**
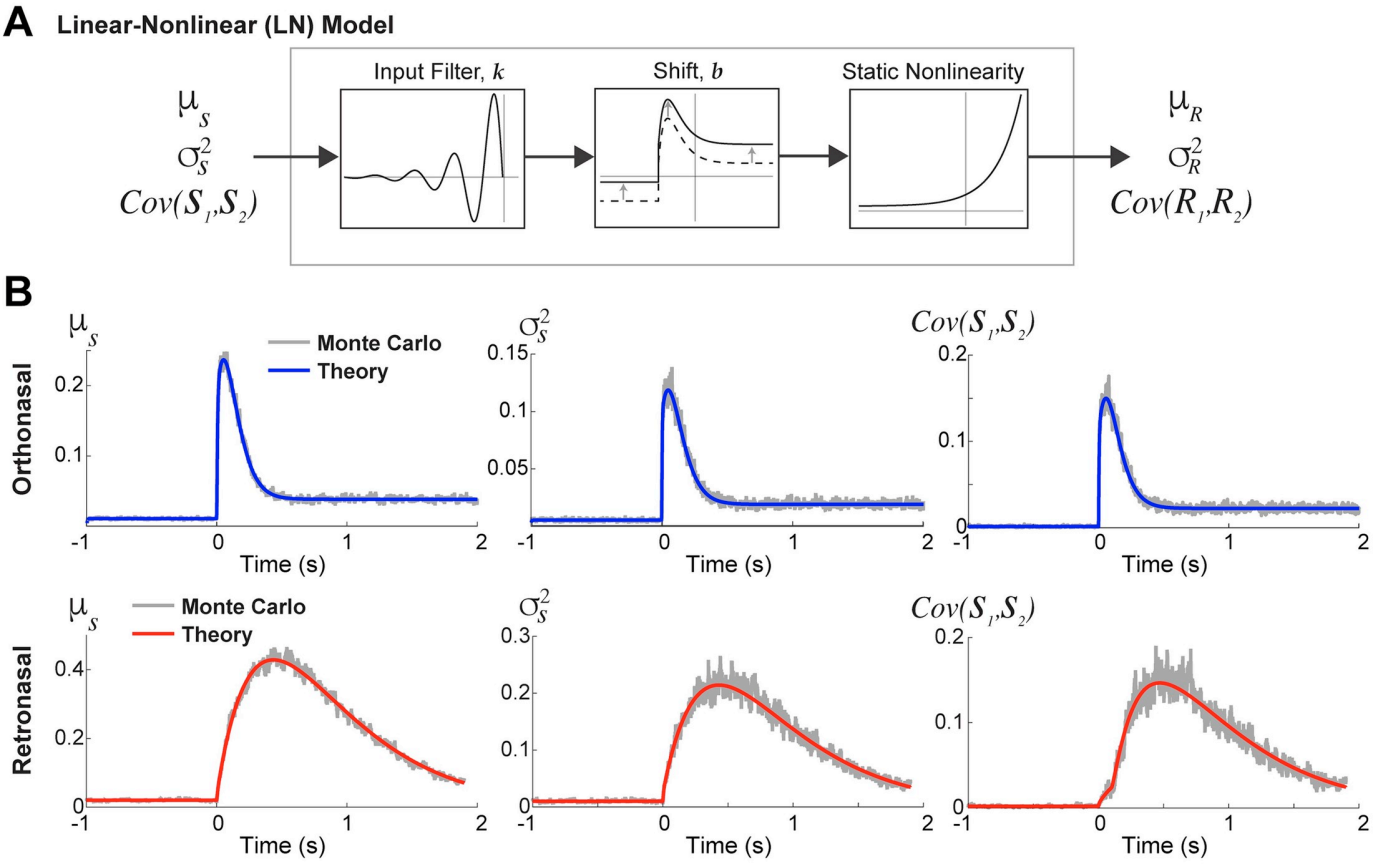
LN framework used to analyze OB transfer of input statistics. **A**) Schematic of the phenomenological linear-nonlinear (LN) model to approximate how the OB network transfers input statistics. **B)** The actual ortho (top row) and retro (bottom row) input synapses used in the biophysical OB model results in Fig 3. Comparisons of the Monte Carlo simulations (Eqs (11) and (12)) and theoretical calculations (Eqs (13), (18) and (22) for respective columns) show smooth curve matches even for correlated time-varying (inhomogeneous) Poisson processes.

We next sought to better understand how our OB network model operates with different ORN inputs. In particular, we investigated whether the same OB network model transfers ortho and retro ORN inputs to MC spike outputs differently or not. We addressed this in a simple and transparent manner with a phenomenological LN model (Fig 4A) to approximate the overall effects of the OB network on ORN inputs. LN-type models have often been used to circumvent the complexities in biophysical spiking models (see [39–41] and Discussion).

#### Description of the LN model

The LN model first applies a linear filter to the input, *X*(*t*), i.e., a convolution with a fixed temporal linear filter *k*, shifts the result by *b*, followed by a static non-linearity (exponential function) to produce an output *Y*(*t*), see Fig 4A:

$$\begin{array}{c} Y\left(t\right)=\text{exp}(\int_{-\infty }^{t}k\left(\tau -t\right)X\left(\tau \right)d\tau +b) \end{array}$$ (1)

**Fig 5:**
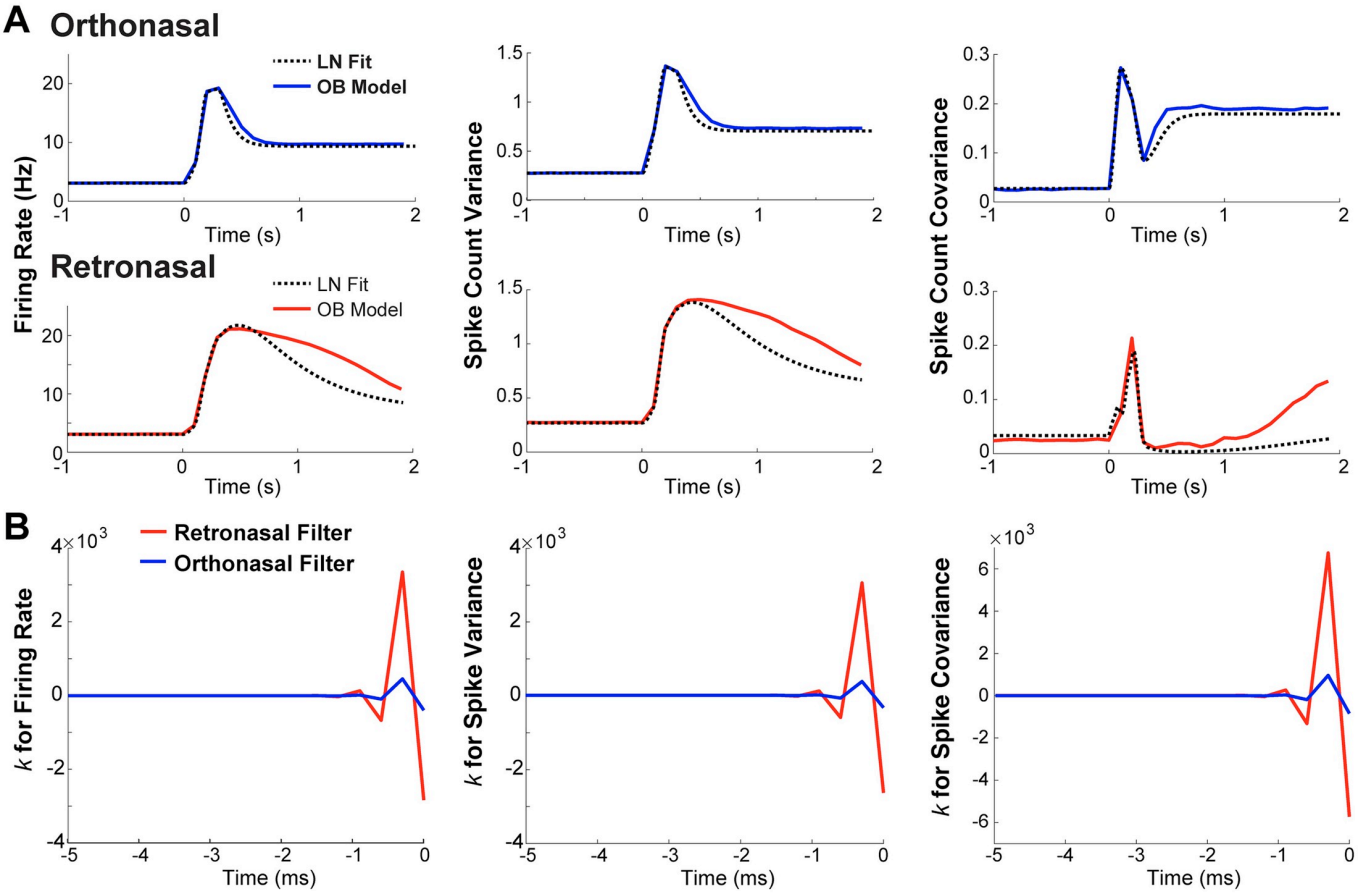
LN model shows that retronasal input results in linear filters with larger magnitudes. **A**) Comparison of LN model output (dashed black curves) to OB network model output statistics for ortho (solid blue curves in top panels) and retro (solid red curves in bottom panels) stimulus with onset at *t* = 0 s. The LN output qualitatively captures OB model output statistics. **B**) Linear filters *k*(*t*) in LN model for ortho (in blue) and retro (in red) stimulus over time (−5 ≤ *t* ≤ 0 ms). Linear filters for retro have larger positive and negative values than with ortho.

For our purposes, $X\left(t\right)\in \{\mu_{S}\left(t\right),\;\sigma_{S}^{2}\left(t\right),\;\text{Cov}\;(S_{1}\left(t\right),S_{2}\left(t\right))\}$ are the statistics of ORN input synapses to the MCs, and *Y*(*t*) is an approximation to the statistics of MC spiking response: $\left\{\text{PSTH}\left(t\right),\;\sigma_{R}^{2}\left(t\right),\;\text{Cov}\;(R_{1}\left(t\right),R_{2}\left(t\right))\right\}$. We calculate *Y*(*t*) (Eq (1)) by minimizing the *L*_2_-norm of the difference between *Y* (*t*) and the simulated MC spike statistic from the biophysical OB model. The LN model is applied separately to each statistic (further details to follow, see Eqs (3)–(5)). For example, for ortho firing rate (Fig 5A, top left), PSTH(*t*) is the blue curve in Fig 5A, top left, the best fit *Y* (*t*) is black dotted curve in Fig 5A (top left), found via:

$$\begin{array}{c} (k(t),b)=\text{arg}\;\underset{k(t),b}{\text{min}}{\left\|Y\left(t\right)-\text{PSTH}\left(t\right)\right\|}_{L_{2}} \end{array}$$ (2)

(also see Materials and methods: **Linear-Nonlinear (LN) model: numerical details**). This procedure is repeated for each statistic and mode of olfaction:

$$\begin{array}{ccc} \mu_{S}\left(t\right) & \underset{\text{LN}}{\to } & \text{PSTH}(t) \end{array}$$ (3)

$$\begin{array}{ccc} \sigma_{S}^{2}\left(t\right) & \underset{\text{LN}}{\to } & \sigma_{R}^{2}\left(t\right),\;(\text{spike}\;\text{count}\;\text{variance})\; \end{array}$$ (4)

$$\begin{array}{ccc} \text{Cov}(S_{1}\left(t\right),S_{2}\left(t\right)) & \underset{\text{LN}}{\to } & \text{Cov}(R_{1}\left(t\right),R_{2}\left(t\right)),\;(\text{spike}\;\text{count}\;\text{covariance})\; \end{array}$$ (5)

That is, we consider different, separate LN models for each statistic, without any mixing effects (e.g., $\sigma_{S}^{2}\left(t\right)$ does not directly affect PSTH(*t*)). Although output statistics generally depend on all input statistics [42–44], we emphasize that our ad-hoc approach here is meant to better understand how the OB model operates *on each statistic* and is not a principled alternative model.

By construction, in the biophysical OB model, both the inputs to each MC and the spike output of each MC have identical marginal statistics, so we are using the LN model to assess how univariate input statistics (mean/var) are mapped to univariate output statistics (mean/var). The covariances depend on 2 variables (bivariate: (*S*_1_, *S*_2_) for input and (*R*_1_, *R*_2_) for output), but the LN model is only for assessing how covariance of inputs maps to covariance of outputs without directly modeling multiple random variables.

For the inputs to the LN model, we use an exact theoretical calculation for $\mu_{S}\left(t\right),\sigma_{S}^{2}\left(t\right),\text{Cov}\;(S_{1}\left(t\right),S_{2}\left(t\right))$ rather than relying on Monte Carlo simulations. The ORN input synapses are driven by correlated time-varying inhomogenous Poisson processes yet we are still able to calculate the first and second order statistics of the ORN inputs in the limit of infinite number of realizations; detailed in Materials and methods: **Calculating time-varying ORN input synapses**, Eqs (13), (18) and (22). A comparison of Monte Carlo simulations of the actual ORN inputs used in our OB model results (Eqs (11) and (12)) to the theoretical calculation (Eqs (13), (18) and (22)) is shown in Fig 4B. We clearly see that the calculations (labeled ‘Theory’) matches all three ORN input statistics with smooth curves, properly accounting for both time-varying ORN input and time-varying input correlation. These calculations do not depend on any asymptotic assumptions; see S4 Fig for more examples.

#### Applying LN models to biophysical OB model results

The LN model is able to fit well to the biophysical OB model output MC spike statistics for both ortho and retro stimuli, shown in Fig 5A. For this reason, we can assume that the LN model provides a decent approximation of how the biophysical OB model transfers the different ORN input statistics. Thus, the resulting linear filters, *k*(*t*) in Fig 5B, succinctly show how the various ORN input statistics are convolved in time by the biophysical OB network model. For all 3 spike statistics, retro input statistics result in filters with larger absolute values (both positive and negative) than ortho, suggesting that the OB network operates in a regime where MC responses are more sensitive to fluctuations with retro input. The resulting *b* values are listed in Table 1; they represent an absolute shift independent of the temporal dynamics. The *b* values are similar for ortho and retro for all statistics except spike count covariance. Although *b* is important for the resulting LN curves (dot-black in Fig 5A), it is not a part of the temporal processing of ORN inputs.

**Table 1:** Parameter *b* for LN model fits to MC spiking statistics in Fig 5.

	PSTH	Variance	Covariance
Orthonasal	2.10	-0.50	-1.56
Retronasal	1.94	-0.55	-3.02

The parameter *b* for the LN model fits (Eq (1)) between orthonasal and retronasal are similar for a given statistic, except for spike count covariance.

### ORN input signatures for ortho/retro

Despite retro eliciting larger firing rates than ortho, the spike count covariance (as well as correlation and Fano factor) with retro stimulation is smaller than with ortho. It has long been known theoretically and experimentally that in uncoupled cells, the spike correlation increases with firing rate (at least with moderate to larger window sizes) [45], in contrast with our data. In coupled networks, the change in correlation with firing rate is complicated and depends on numerous factors [21–24]. Thus, the components of ORN inputs that result in these differences (higher firing and less covariance for retro than with ortho) in the same OB network are not obvious.

**Fig 6:**
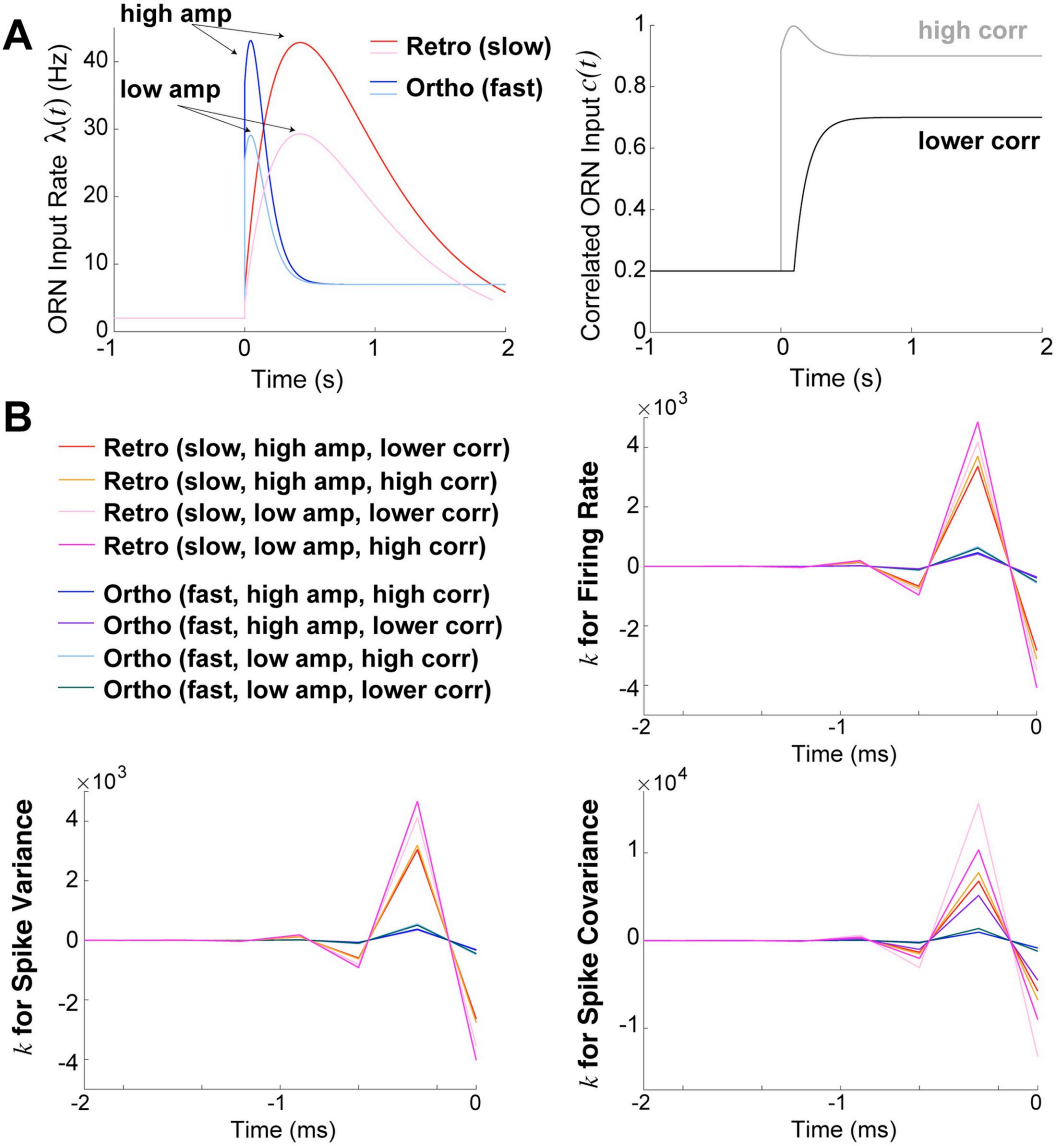
Temporal profile is crucial for larger magnitude filters. **A**) Different combinations of input rates (left) including slower increase and decay (retro-like) and faster increase and decay (ortho-like) as well as high and low amplitude as labelled. Two different input correlations (right), with high correlation in gray, and lower correlation in black. **B**) Resulting linear filters *k*(*t*) have consistently larger absolute values when temporal profile of ORN inputs is slower (retro-like), compared to faster (ortho-like).

So we use our computational framework to uncover the important feature(s) of ORN input that: i) results in MC spike statistics consistent with our salient data trends, and ii) linearly filters ORN inputs with larger values with retro than with ortho input. Here we disregard the biological differences in ortho and retro ORN inputs to consider 3 core attributes of ORN inputs that influence how the biophysical OB model operates:

- Temporal (faster increase and decay, or slower increase and decay; see Fig 6A, left)
- Amplitude (low or high, see Fig 6A, left)
- Input correlation (lower or higher, black and gray curves respectively, in Fig 6A, right)

We consider a total of 8 different ORN input profiles consisting of various combinations of amplitude, input correlation, temporal profiles. The LN model fit to the OB model (i.e., MC spike statistics) for these 8 different ORN input profiles are all similar, well approximating how the OB coupled network transfers input statistics (see Fig 5A and S5 Fig). Fig 6B clearly shows that the slower increase and decay in input rate (redish/lighter) consistently results in linear filters *k*(*t*) with larger absolute values than with faster increase/decay (bluish/darker). The larger filter values holds with all 3 statistics, and with all variations of amplitude and input correlation. Thus, the OB network consistently has filters with larger absolute values when the input profile is slower (i.e., retronasal-like). The resulting LN model *b* values are listed in Table 2 for reference, although these values represent an absolute scaling independent of the temporal dynamics.

**Table 2:** Parameter *b* for LN model fits to MC spiking statistics in Fig 6.

Temporal	Amplitude	ORN correlation	PSTH	Variance	Covariance
Fast (ortho)	**High**	**High**	**2.10**	**-0.50**	**-1.56**
	High	Low	1.99	-0.56	-1.35
	Low	High	2.03	-0.55	-1.62
	Low	Low	1.97	-0.61	-3.42
Slow (retro)	High	High	2.04	-0.50	-1.11
	**High**	**Low**	**1.94**	**-0.55**	**-3.02**
	Low	High	1.70	-0.72	-1.36
	Low	Low	1.59	-0.81	-3.18

The parameter *b* for the LN model fits (Eq (1)) of the various parameters for temporal profile, amplitude, and input correlation. Amplitude and ORN input correlation profiles as defined for Figs 3–5 and associated values previously listed in Table 1 are noted in bold.

**Fig 7:**
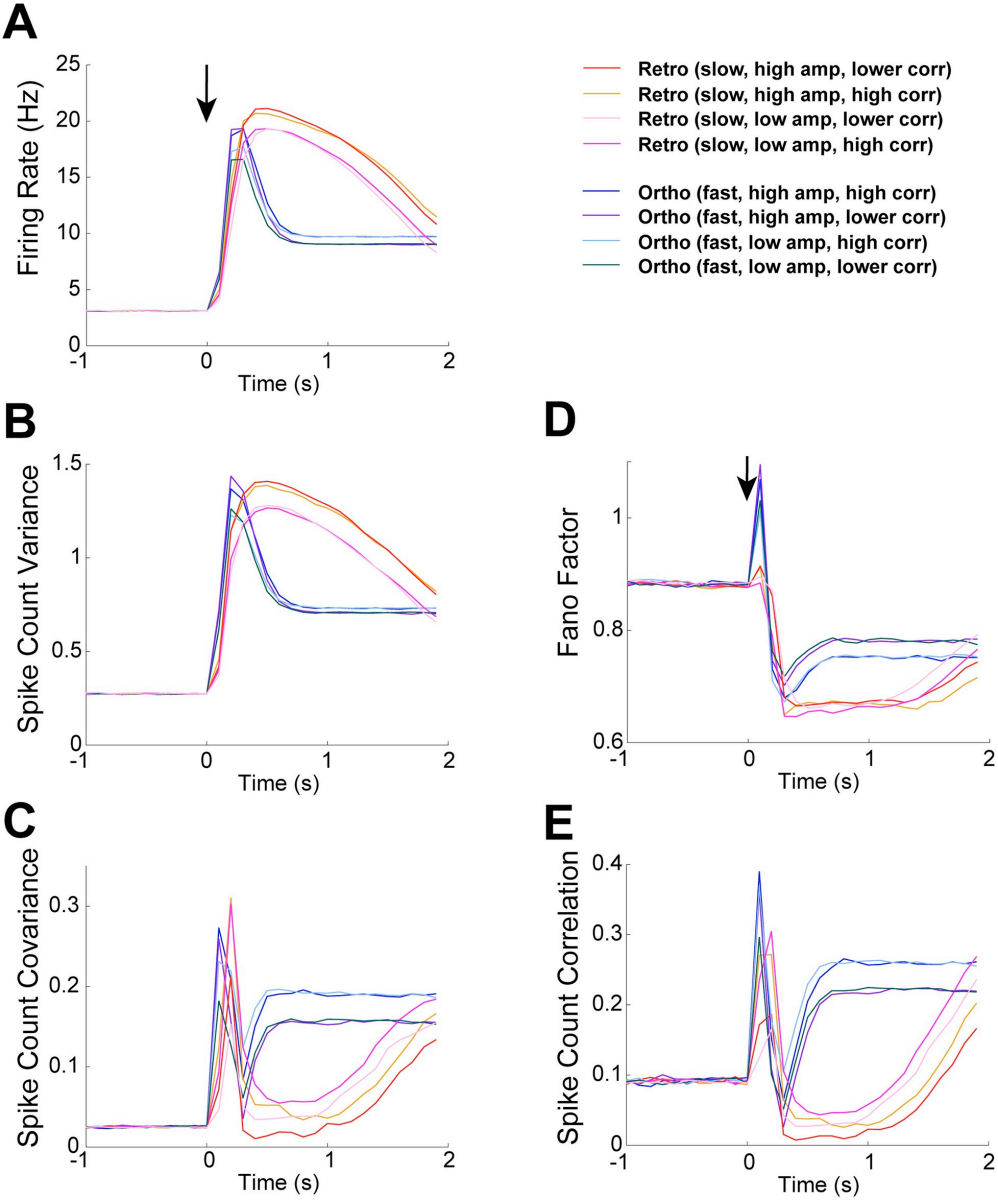
Comparison of all 8 OB model results. The 8 different OB model results are from varying temporal profile, amplitude height, and input correlation (2 ways each, see Fig 6A). Different temporal profiles is key for both having different model spike statistics **and** for best matching qualitative differences in our data (see Fig 3). **A**) Firing rate in Hz (left) is slightly lower with low input rate amplitude, but no significant difference with different input correlations. **B**) Spike count variance, similar to firing rate, has only slightly lower values with low input rate amplitude. **C**) Spike count covariance is lower with lower input correlation for all of ortho evoked state (not surprisingly). However, retro (fast) with lower amplitude steadily increases above higher amplitude after about 1 s in the evoked state. **D**) Fano Factor model results only change modestly. **E**) Pearson’s spike count correlation, similar to spike count covariance, is lower with lower input correlation and similarly for retro (fast), there is an increase with higher input correlation.

Fig 7 shows all 8 OB model results for each spike statistic. For all first and second order statistics, including scaled measures of variability, the most distinct attribute that distinguishes our model results is the temporal profile of input. Importantly, the temporal profile is the key attribute to best capture the differences in ortho and retro our experimental data (see Fig 3). The slow increase and decay in input rate consistently results in retro-like spiking trends while the fast increase and decay in input rate results in ortho-like spiking trends. Thus, our models show that the temporal profile is a signature of retro and ortho stimulation, and emphasizes the critical role of ORN inputs for shaping how the same OB network modulates ortho and retro stimuli.

## Discussion

We investigated how odors processed via the orthonasal and retronasal routes result in different OB spike statistics, analyzing in detail how ORN inputs transfer to MC spike outputs. Motivated by our *in vivo* rat recordings that showed significant differences in first and second order spiking statistics of MC, we developed a realistic OB network model to investigate the dynamics of stimulus-evoked spike statistic modulation (higher firing and lower covariance/correlation with retro than with ortho). Our OB model balances biophysical attributes [29, 30] with computational efficiency. The OB model is able to capture salient trends in our data with both ortho and retro stimulation, and should be useful for future studies of OB. We successfully used the biophysical OB model, paired with a phenomenological LN model, to analyze how different ORN inputs lead to different dynamic transfer of input statistics. We also showed that the temporal profile of ORN inputs is a key determinant of ortho versus retro input via model matching our data. The output spike statistics are crucial because the OB relays odor information to higher cortical regions, and thus our work may have implications for odor processing with different modes of olfaction [9–11].

To the best of our knowledge, our experiments detail the differences in MC spiking with ortho and retro stimuli for the first time. However, the work of Scott et al. [19] is related; they used 4 electrodes to record OB spiking activity in the superficial layers of OB in rats. Their results are difficult to directly compare to ours as they focus on superficial OB in the epithelium rather than the mitral cell layer, but at least the trial-averaged firing rates in their data appear to be consistent with our data. Moreover, our multi-electrode array recordings enable us to consider trial-to-trial covariance of spiking.

The key attribute(s) of ORN inputs that can result in different ortho and retro MC spike statistics consistent with our data are not obvious. Indeed, retro stimulation resulted in larger firing rates than ortho, and the spike count covariance (as well as correlation and Fano factor) with retro stimulation is smaller than with ortho, in contrast to uncoupled cells where correlation increases with firing rate [45]. Using various models, we were able to consider how three components of ORN inputs (temporal profile, amplitude, and input correlation) result in different OB dynamics with regards to transferring input statistics to outputs. Prior experiments [11, 16, 17] have shown these input components can differ with ortho and retro inputs. We found that the temporal profile (fast versus slow) plays a critical role for both capturing our data and for shaping the transfer of inputs to outputs, i.e., retro inputs consistently resulted in larger temporal filter values, so the OB network is more sensitive to fluctuations of retro input statistics than ortho. To capture the salient trends in our data, we find slower input rate (rise and decay) is a key signature of retronasal stimulation, while faster rise and decay [11, 17, 19] is similarly a key signature of orthonasal stimulus.

The temporal differences between ortho versus retro have previously been thought to play a role in distinguishing ortho/retro stimulation at the ORN [11, 16, 17, 19, 20], but whether this carried over to the OB and if this held at the level of spiking was unknown. Here we demonstrate the importance of different temporal input to OB for ortho versus retro.

We used an ad-hoc LN model framework because many of biological complexities are removed yet important features are retained. That is, neurons are known to linearly filter inputs, and spike generation is inherently nonlinear, i.e., finding linear filters of neurons is not new [41], and they are related to the spike-triggered average [46]. Thus, LN-type models have been used in many contexts, often to circumvent biophysical modeling, and most notably with generalized linear models [47, 48] (also see [40]) where various filters (stimulus, post-spike) and model components are fit to data using maximum likelihood. Connecting the large gap between biophysical models and LN models is daunting, but see Ostojic and Brunel [39] who relate stochastic integrate-and-fire type models to LN. Our approach here is much simpler than the aforementioned works because we simply wanted to assess how a particular statistic (mean, variance or covariance) mapped via the OB network model in a simple and transparent manner. An enhanced data-driven approach to fitting an LN-type model that relies on experimental data of *both* ORN inputs and MC spike outputs with many trials might better reveal differences in how the OB operates with ortho versus retro. However, we currently do not know if such a dataset exists.

Here we list some limitations of our study. We only considered the MC response to a single food odor despite a large variety of food (and non-food) odors animals encounter. Different odors activate different olfactory receptors that could lead to qualitatively different population MC spiking activity than what we report here. Retronasal odors are predominately food odors, and studies have shown that humans can more accurately detect food odors (vs. non-food odors) delivered retronasally [10]. Frasnelli et al. [49] showed that food versus non-food odors illicit varying neural responses in humans when introduced ortho- versus retronasally. An fMRI BOLD study showed that cortical activity in humans differed when odors were introduced via the ortho or retro routes, specifically with food odors [9]. Thus our choice of a food odor is a natural first step for investigating retronasal MC responses. Also, we attributed the differences in ortho/retro MC responses solely to ORN inputs when in fact many regions synapse to OB [50]. For example, optogenetic studies [51, 52] have shown that feedback from olfactory cortex to OB is relatively strong and inhibition dominated. Whether this cortical feedback (or other external modulation) differs for ortho and retro stimulation is currently unknown. Moreover, odor-specific cortical feedback to OB [53] could alter the OB spike correlation, a factor that our modeling study did not account for. Finally, our data is from anaesthetized rats that enabled control of odor delivery and excluded confounding factors such as the breath cycle and sniff rate [54, 55]. However, the MC spike activity in awake rodents can be quite different than in anesthetized [56], so whether our reported differences in ortho versus retro MC spiking hold in awake rodents is an open question. We hope our work here inspires more research into the differences between ortho versus retro olfaction, in particular in downstream olfactory circuits and with other experimental preparations.

With a combination of experiments and different scales of neural network modeling, we provide a basis for understanding how differences in OB spiking statistics arise with these 2 natural modes of olfaction. More generally, our model framework provides a road map for how to analyze attributes responsible for different OB spiking when driven by differences in ORN inputs.

## Materials and methods

### Ethics statement

All procedures were carried out in accordance with the recommendations in the Guide for the Care and Use of Laboratory Animals of the National Institutes of Health and approved by University of Arkansas Institutional Animal Care and Use Committee (protocol #14049). Isoflurane and urethane anesthesia were used and urethane overdose was used for euthanasia.

### Code availability

See https://github.com/michellecraft64/OB for MATLAB code implementing the single-compartment biophysical model, the equations for synaptic input statistics, and the linear-nonlinear (LN) model.

### Single-compartment biophysical OB model

Models of all three cell types (MC, PGC, GC) are based on models developed by the Cleland Lab [29, 30]. We consider two glomeruli each with a representative MC, PGC, GC (see Fig 2B). Each cell is a conductance-based model with intrinsic ionic currents. The voltage responses of all three cell types, measured in experiments and in a multi-compartment model [29, 30], are generally captured in our single-compartmental model, see Fig 2A. Here we describe all of the pertinent model details thoroughly; for other extraneous details and implementation, please refer to provided code on GitHub.

#### Individual cell model

$$\begin{array}{c} C_{j}\frac{dV_{j}}{dt}=I_{j,\text{App}}-\sum I_{j,\text{Ion}}-\sum I_{j,\text{Synapse}}-\sum I_{j,\text{ORN}}, \end{array}$$ (6)

The voltages of all model cells are governed by a Hodgkin-Huxley type current balance equation (Eq (6) above for the *j*^th^ cell) consisting of voltage (*V*), membrane capacitance (*C*), applied current (*I*_App_), ionic currents (*I*_Ion_), synaptic currents (*I*_Synapse_), and ORN inputs (*I*_ORN_); see Tables 3 and 4 for units and numerical values, respectively. For our modeling purposes, the ionic currents and the ORN inputs are modified from [29, 30] and described below.

**Table 3:** Description of model parameters.

**Resistance and Capacitance**	
**Variable**	**Description**
*R*_m_	Membrane Resistance (KΩ-cm^2^)
*C*_m_	Membrane Capacitance (*μ*F/cm^2^)
*R*_a_	Cytoplasmic (Axial) Resistance (Ω-cm)
**Ionic Currents (*μ*A/cm^2^)**	
**Variable**	**Description**
*I*_Na_	Fast, Spike-Generating Sodium Current
*I*_NaP_	Persistent Sodium Current
*I*_DR_	Potassium Delayed Rectifier
*I*_A_	Fast-Activating Transient Potassium Current
*I*_M_	Noninactivating Muscarinic Potassium Current
*I*_KS_	Slow-Inactivating Transient Potassium Current
*I*_H_	Hyperpolarization-Activated Current
*I*_CaL_	L-type Calcium Current
*I*_CaP/N_	High-Threshold Calcium Current
*I*_CaT_	Low-Threshold Inactivating Calcium Current
*I*_CAN_	Ca^2+^-Activated Nonspecific Cation Current
*I*_KCa_	Ca^2+^-Dependent Potassium Current
**Reversal Potentials**	
**Variable**	**Description**
*E*_L_	Leak Current Reversal Potential
*E*_Na_	Sodium Reversal Potential
*E*_K_	Potassium Reversal Potential
*E*_H_	Hyperpolarization-Activated Reversal Potential
*E*_cation_	Ca^2+^-Activated Nonspecific Cation Reversal Potential
*E*_Ca_	Calcium Reversal Potential
**Calcium Dynamics**	
**Variable**	**Description**
*w*	Perimembrane Thickness
*z*	Ca^2+^ Ion Valence
*F*	Faraday Constant
*τ*_Ca_	Ca^2+^ Removal Rate
[Ca^2+^]	Intracellular Ca^2+^ Concentration
[Ca^2+^]_rest_	Ca^2+^ Resting Concentration

**Table 4:** Parameter values for each cell type. Each of these values are the same as defined by [30] with the exception of maximal conductance values which are the sum of all cell compartments (soma, dendrite, axon, etc.) as defined by [30]. Additionally, any conductance value denoted by − implies that this ionic current is not included in the associated cell.

**Resistance and Capacitance**			
**Variable**	**MC Value**	**GC Value**	**PGC Value**
*R*_m_	30	30	20
*C*_m_	1.2	2.0	1.2
*R*_a_	70	70	80
**Maximal Conductance (mS/cm^2^)**			
**Variable**	**MC Value**	**GC Value**	**PGC Value**
*g*_Na_	120	70	70
*g*_NaP_	0.42	—	—
*g*_DR_	70	25	25
*g*_A_	10	80	40
*g*_M_	—	0.5	1.0
*g*_KS_	84	—	—
*g*_H_	—	—	0.2
*g*_CaL_	0.85	—	—
*g*_CaP/N_	—	0.2	1.0
*g*_CaT_	—	0.1	3.0
*g*_CAN_	—	1.0	—
*g*_KCa_	5	0.5	2.0
**Reversal Potentials (mV)**			
**Variable**	**MC Value**	**GC Value**	**PGC Value**
*E*_L_	-60	-60	-65
*E*_Na_	45	45	45
*E*_K_	-80	-80	-80
*E*_H_	0	0	0
*E*_cation_	10	10	10
**Calcium Dynamics**			
**Variable**	**MC Value**	**GC Value**	**PGC Value**
*w*	1 *μ*m	0.2 *μ*m	0.2 *μ*m
*z*	2	2	2
*τ*_Ca_	10 ms	800 ms	800 ms
[Ca^2+^]	dynamic	dynamic	dynamic
[Ca^2+^]_rest_	0.05 *μ*mol/1	0.05 *μ*mol/1	0.05 *μ*mol/1

#### Ionic currents

$$\begin{array}{c} I_{i}=g_{i}m^{p}h^{q}\left(V-E_{i}\right), \end{array}$$ (7)

The ionic currents are defined by Eq (7) above (for specific ion type *i*) and account for maximal conductance (*g*), activation variable (*m*) with exponent (*p*), inactivation variable (*h*) with exponent (*q*), time-varying voltage (*V* assumed to be isopotential), and reversal potential (*E*_*i*_). All parameters and function for intrinsic ionic currents and their gating variables are the same as in [29, 30] with the exception of maximal conductance. We chose to condense the model as defined in [29, 30] by collapsing all compartments to a single-compartment, and we set the maximal conductance as the sum of all maximal conductance values (e.g., in PGC, *I*_Na_ has maximal conductance *g*_Na_ = 70 mS/cm^2^ because [29] set *g*_Na_ = 50 mS/cm^2^ in the soma and *g*_Na_ = 20 mS/cm^2^ in the spine). All summed maximal conductance values used are listed for reference in Table 4. The calcium dynamics used to define the calcium-related ionic currents are the same as in [29, 30].

#### Synaptic currents

$$\begin{array}{c} I_{\text{syn}}=wg_{\text{syn}}sB\left(V\right)\left(V-E_{\text{syn}}\right), \end{array}$$ (8)

$$\begin{array}{c} \frac{ds}{dt}=\alpha F\left(V_{\text{pre}}\right)\left(1-s\right)+\beta s, \end{array}$$ (9)

Eqs (8) and (9) are the equations for the synaptic variables, where all presynaptic GCs and PGCs provide GABA_A_ inputs, and all presynaptic MCs provide both AMPA and NMDA inputs. *B*(*V*) in Eq (8) is the NMDA-specific magnesium block function (*B*(*V*) = 1 for all other synapses), and *s*(*t*) is the fraction of open synaptic channels. The channel opening rate constants (*α* and *β*) are normalized sigmoidal function of presynaptic membrane potential (*F*(*V*_pre_) in Eq (9)), the same as in [29, 30]. We also define the conductance parameter (*g*_syn_) and reversal potentials (*E*_syn_) as [29, 30] have, with *g*_GABA_ = 1.5 nS for GC→MC synapses, *g*_GABA_ = 2 nS for PGC→MC synapses, *g*_AMPA_ = 2 nS and *g*_NMDA_ = 1 nS for both MC→PGC and MC→GC synapses; *E*_syn_ = 0 mV for AMPA and NMDA currents, and *E*_syn_ = −80 mV for GABA_A_ currents.

#### ORN input

$$\begin{array}{c} I_{\text{ORN}}=S\left(t\right)\left(V-E_{X}\right), \end{array}$$ (10)

$$\begin{array}{c} \tau_{X}\frac{dS}{dt}=-S+a_{X}\tau_{X}\sum_{j}\delta \left(t-t_{k}\right), \end{array}$$ (11)

The ORN inputs for each cell consist of both excitatory and inhibitory inputs as specified in Eqs (10) and (11) where *X* ∈ {*E*, *I*}. The reversal potential value (*E*_*X*_) is much larger for excitatory inputs and smaller for inhibitory. The function $S\left(t_{k}^{+}\right)=S\left(t_{k}^{-}\right)+a_{X}$ accounts for the random times (*t*_*k*_) when *S* instantaneously increases by *a*_*X*_. The random times, *t*_*k*_, are governed by an inhomogeneous Poisson process with rate λ_*X*_(*t*). This aligns with experimental evidence that ORN spiking is Poisson-like in the spontaneous state [27]. Thus, we extend the notion that ORN spiking would be Poisson-like in the evoked state with increased rate λ_*X*_(*t*) varying in time. Finally, we set the synaptic rise and decay time constants (*τ*_*X*_) to be 5.5 ms for PGCs and GCs, 10 ms for MCs, as in [29, 30].

The ORN input rates can be pairwise correlated, which is achieved by the parameter *c*_*j*,*k*_ ∈ [0, 1], for cells *j* and *k* detailed by Eq (12) below:

$$\begin{array}{c} \lambda_{j}\left(t\right)={\tilde{\lambda }}_{j}\left(t\right)-\bar{\lambda }\left(t\right)c_{j,k}\left(t\right). \end{array}$$ (12)

where ${\tilde{\lambda }}_{j}\left(t\right)$ and ${\tilde{\lambda }}_{k}\left(t\right)$ are the individually defined ORN input rates for cells *j* and *k*, and $\bar{\lambda }\left(t\right)≔\text{min}\left({\tilde{\lambda }}_{j}\left(t\right),{\tilde{\lambda }}_{k}\left(t\right)\right)$.

### Fitting biophysical network model to data

The biophysical OB model described thus far was adopted directly from Li & Cleland, aside from our single-compartment simplification where we lumped all ionic currents into one compartment and used the sum of the (maximal) conductances from all compartments. Here we describe how the network model was tuned to capture the salient features of our experimental data. We did not systematically consider large volumes of parameter space due to the enormous computational resources required for 50,000 simulations of the model for each parameter set to accurately simulate the spike count statistics. After model parameters were set, the only manual tuning we did was to consider several Poisson input rates λ_*O*/*R*_(*t*) (see Eqs (10)–(12)) for the ORN input synapses (see S3 Fig)—even the ORN input correlations *c*_*j*,*k*_(*t*) that we arbitrarily chose were fixed throughout.

Note that we did not further tune the intrinsic properties of the individual cells; the PGC, MC, and GC parameters are as stated above with behavior shown in Fig 2A.

#### Specifying coupling strengths

We used the same equations for the synaptic variables as Cleland [30], but set the coupling strengths *w* (see Eq (8)) to: *w*_*M*←*G*_ = 3 (independent inhibition), *w*_*M*←*Gc*_ = 0.3 (common inhibition to MC), *w*_*G*←*M*_ = 1 (same for both AMPA, NMDA), *w*_*Gc*←*M*_ = 0.5 (inhibition to common GC), *w*_*P*←*M*_ = 1 and *w*_*M*←*P*_ = 2 (same for both AMPA, NMDA). These coupling strengths were chosen in part from results in Ly et. al [57] who used a related/simpler OB network model with the same 2 glomeruli architecture to find regions of parameter space that best fit orthonasal experimental data. Similar to Ly et. al [57] (see their Figs 2, 3, and 6) we set independent inhibition from GC to MC to be greater than excitation from MC to GC, and shared GC inhibition to MC to be relatively weak (i.e., *w*_*Gc*←*M*_ ≤ *w*_*G*←*M*_ ≤ *w*_*M*←*G*_). The coupling strengths were never tuned, they were fixed throughout.

#### Specifying ORN input

The ORN inputs (Eqs (10)–(12)) consists of a Poisson input rate, and input correlation between pairs of cells. We set the correlation (*c*_*j*,*k*_) between the following cell pairs: MC and PGC pair within a glomerulus have *c*_*j*,*k*_ = 0.3 because they receive inputs from the same ORN cells; the two MCs have correlated ORN input [58] (*c*_*j*,*k*_(*t*) time-varying as in Fig 2Cii); and between all 3 GCs because they are known to synchronize [30, 59] (*c*_*j*,*k*_ = 0.3 for all 3 different pairs of GCs). All other pairs of cells have no ORN input correlation. Note that input correlation for the 2 MCs increased with odor to mimic increased correlation of glomeruli activity. In Fig 2Cii, input correlation for the 2 MCs are constrained such that *c*_*R*_(*t*) < *c*_*O*_(*t*). This is based on prior imaging studies that show retronasal stimulation activates spatially smaller regions of glomeruli inputs than orthonasal, and that the activation regions from retro are subsets of ortho [11, 17]. For specific algebraic formula of *c*_*R*/*O*_(*t*), please refer to code on GitHub.

We considered several different λ_*O*/*R*_(*t*), the inhomogeneous Poisson input rate of *t*_*k*_ in Eq (11) (with constraints described below) and chose the ones that best matched the time-varying firing rates (Fig 7A). The ortho- vs. retronasal odor input rates, λ_*O*/*R*_(*t*), are constrained such that λ_*O*_(*t*) increases faster and more abruptly than λ_*R*_(*t*) with odor, and λ_*R*_(*t*) decays slower than λ_*O*_(*t*); this is all based on ORN imaging studies [11, 17]. Inputs consist of both excitatory synapses (with rate λ_*O*/*R*_(*t*)) and inhibitory synapses (with rate 0.75λ_*O*/*R*_(*t*)) to capture other unmodeled inhibitory effects.

To first understand how MC firing rates depends on λ(*t*) without any consideration for ortho or retro, we used a simple alpha-function form in the evoked state: λ(*t*) = *te*^−*t*/*τ*^, surveying 6 different *τ* (see S3(A) Fig, left). The resulting MC firing rates (S3(A) Fig, right with 2,000 realizations) was informative for how to manually set the input values (spontaneous, peaked-evoked, etc.). S3(B) Fig shows all of the λ_*O*/*R*_(*t*) we tried, notice that they all satisfy the constraints described above. Via trial and error with 2,000 realizations, we only looked at the resulting firing rates (PSTH), insuring the simulations matched the ortho data well. We were fortunate in fitting the retro firing rate data, trying only 2 λ_*R*_(*t*). The other spike statistics (e.g., covariance, Fano factor) were never accounted for in our consideration of different λ_*O*/*R*_(*t*), which is perhaps why the fit to the spike covariance data is so bad.

### Calculating time-varying ORN input statistics of synapses

Here we describe a method to capture the effects of ORN input statistics of synapses to the biophysical OB model, in the limit of infinite realizations. These methods are very useful as inputs for the LN model, without which one would have to use averages from Monte Carlo simulations that contain deviations from finite size effects. Taking the expected value of Eq (11) results in an equation for the average of *S*(*t*), *μ*_*S*_(*t*):

$$\begin{array}{c} \tau_{X}\mu_{S}\left(t\right)=-\mu_{S}+\tau_{X}a_{X}\lambda \left(t\right), \end{array}$$ (13)

To derive the equation for variance $\sigma_{S}^{2}\left(t\right)$, we multiply Eq (11) by itself. We can equivalently rewrite Eq (11) as an integral:

$$\begin{array}{c} S\left(t\right)=a_{X}\int_{-\infty }^{t}e^{-\left(t-u\right)/\tau_{X}}D\left(u\right)\;du, \end{array}$$ (14)

where $D\left(t\right)≔\sum_{j}\delta \left(t-t_{k}\right)$. So

$$\begin{array}{c} S^{2}\left(t\right)={\left(a_{X}\right)}^{2}\int_{-\infty }^{t}\int_{-\infty }^{t}D\left(u\right)D\left(v\right)e^{-\left(t-u\right)/\tau_{X}}e^{-\left(t-v\right)/\tau_{X}}\;dudv \end{array}$$ (15)

Recall that E[D(u)D(v)]=λ(v)δ(u−v)+λ(u)λ(v), so we have:

$$\begin{array}{ccc} E[S^{2}\left(t\right)] & = & {\left(a_{X}\right)}^{2}\int_{-\infty }^{t}\lambda \left(v\right)e^{-2\left(t-v\right)/\tau_{X}}\;dv+{\left(\mu_{S}\left(t\right)\right)}^{2} \end{array}$$ (16)

$$\begin{array}{ccc} \Rightarrow \sigma_{S}^{2}\left(t\right) & = & {\left(a_{X}\right)}^{2}\int_{-\infty }^{t}\lambda \left(v\right)e^{-2\left(t-v\right)/\tau_{X}}\;dv, \end{array}$$ (17)

Equivalently, $\sigma_{S}^{2}\left(t\right)$ satisfies the ODE:

$$\begin{array}{c} \tau_{X}\frac{d\sigma_{S}^{2}\left(t\right)}{dt}=-2\sigma_{S}^{2}+\tau_{X}{\left(a_{X}\right)}^{2}\lambda \left(t\right), \end{array}$$ (18)

Similarly for *S*_*j*_(*t*)*S*_*k*_(*t*) correlated synapses, we have:

$$\begin{array}{c} S_{j}\left(t\right)S_{k}\left(t\right)=(a_{X_{j}}a_{X_{k}})\int_{-\infty }^{t}\int_{-\infty }^{t}D\left(u\right)D\left(v\right)e^{-\left(t-u\right)/\tau_{X_{j}}}e^{-\left(t-v\right)/\tau_{X_{k}}}\;dudv, \end{array}$$ (19)

By our model construction $E[D(u)D(v)]=c_{j,k}\left(v\right)\bar{\lambda }\left(v\right)\delta \left(u-v\right)+\lambda_{j}\left(u\right)\lambda_{k}\left(v\right)$, where $\bar{\lambda }\left(t\right)≔\min(\lambda_{j}\left(t\right),\lambda_{k}\left(t\right))$, so we have:

$$\begin{array}{ccc} E[S_{j}\left(t\right)S_{k}\left(t\right)] & = & (a_{X_{j}}a_{X_{k}})\int_{-\infty }^{t}c_{j,k}\left(v\right)\bar{\lambda }\left(v\right)e^{\left(-\tau_{X_{j}}-\tau_{X_{k}}\right)\left(t-v\right)/\left(\tau_{X_{j}}\tau_{X_{k}}\right)}\;dv+\mu_{S_{j}}\left(t\right)\mu_{S_{k}}\left(t\right) \end{array}$$ (20)

$$\begin{array}{ccc} \Rightarrow \text{Cov}(S_{j}\left(t\right),S_{k}\left(t\right)) & = & (a_{X_{j}}a_{X_{k}})\int_{-\infty }^{t}c_{j,k}\left(v\right)\bar{\lambda }\left(v\right)e^{\left(-\tau_{X_{j}}-\tau_{X_{k}}\right)\left(t-v\right)/\left(\tau_{X_{j}}\tau_{X_{k}}\right)}\;dv, \end{array}$$ (21)

Cov(*S*_*j*_(*t*), *S*_*k*_(*t*)) equivalently satisfies the ODE:

$$\begin{array}{c} \tau_{X_{j}}\tau_{X_{k}}\frac{d\text{Cov}(S_{j}\left(t\right),S_{k}\left(t\right))}{dt}=-\left(\tau_{X_{j}}+\tau_{X_{k}}\right)\text{Cov}\left(S_{j}\left(t\right),S_{k}\left(t\right)\right)+\left(\tau_{X_{j}}\tau_{X_{k}}\right)(a_{X_{j}}a_{X_{k}})c_{j,k}\left(t\right)\bar{\lambda }\left(t\right), \end{array}$$ (22)

The calculations for the dynamic (time-varying) synapse statistics are important for capturing realistic statistics because a steady-state approximation can be very inaccurate, especially when the time-varying correlation and ORN spiking rate change quickly relative to the time-scales (*τ*_*X*_). The quasi-steady-state approximation is:

$$\begin{array}{ccc} \mu_{S}\left(t\right) & \approx & \tau_{X}a_{X}\lambda \left(t\right) \end{array}$$ (23)

$$\begin{array}{ccc} \sigma_{S}^{2}\left(t\right) & \approx & \frac{\tau_{X}{\left(a_{X}\right)}^{2}\lambda \left(t\right)}{2} \end{array}$$ (24)

$$\begin{array}{ccc} \text{Cov}(S_{j}\left(t\right),S_{k}\left(t\right)) & \approx & \frac{\tau_{X_{j}}\tau_{X_{k}}}{\tau_{X_{j}}+\tau_{X_{k}}}a_{X_{j}}a_{X_{k}}c_{j,k}\left(t\right)\bar{\lambda }\left(t\right) \end{array}$$ (25)

S4 Fig shows several more examples demonstrating the accuracy of the calculations (Eqs (13), (18) and (22)) and how inaccurate Eqs (23)–(25) can be.

### Linear-Nonlinear (LN) model: Numerical details

We use the above described ODEs (Eqs (13), (18), and (22)) to simplify the calculations of ORN input statistics for use with the LN model framework. Previous work has implemented LN-type models as an alternative to biophysical spiking models with various conditions (see [39–41] and Discussion). Fig 4A illustrates a schematic of the LN model parameters, linear filter (*k*) and shift (*b*), that are used with the ORN input statistics ($X\in \{\mu_{S},\;\sigma_{S}^{2},\;\text{Cov}\left(S_{1},S_{2}\right)\}$) in order to construct an approximation (*Y*) to the biophysical OB network model’s output MC spike statistics (PSTH(*t*), $\sigma_{R}^{2}$, Cov(*R*_1_, *R*_2_)). The LN model is summarized as:

$$\begin{array}{c} Y\left(t\right)=f(\int_{-\infty }^{t}k\left(\tau -t\right)X\left(\tau \right)d\tau +b) \end{array}$$ (26)

Where we define our function *f* as an exponential, and we can approximate the integral numerically as follows:

$$\begin{array}{c} \int_{-\infty }^{t}k\left(\tau -t\right)X\left(\tau \right)d\tau +b\approx \sum_{l=0}^{n-1}\vec{k}\left(l\right)\vec{X}\left(j-l\right)\;\Delta \tau +\vec{b} \end{array}$$ (27)

Where *n* denotes the number of time points included in the linear filter, and *j* denotes the points in time of input statistic $\vec{X}$ of size Lt. Then, we can rewrite Eq (27) in matrix vector form $A\vec{w}=\vec{v}$ where: *A* is the Toeplitz matrix of size (Lt − *n* + 1) × *n* of our input statistic ($\vec{X}$) with an additional row of value one to account for shift; $\vec{w}$ is our linear filter ($\vec{k}$) and shift ($\vec{b}$); and $\vec{v}$ is our OB network MC spike statistic to which we fit our filter. Then, we solve for $\vec{w}$ using Least Squares approximation by QR decomposition. The linear filter ($\vec{k}$) converges to 0 by construction, therefore we truncate the filter at −0.1 s and set *k* = 0 for the remaining time −1 ≤ *t* < −0.1. Then, the LN output approximation (*Y*) of the MC spike statistic is calculated as follows:

$$\begin{array}{c} Y\left(t\right)=f(K\cdot \vec{X}+\vec{b}) \end{array}$$ (28)

Where *K* denotes the convolutional matrix constructed from the truncated linear filter *k*.

### Electrophysiological recordings

We decided to use recordings from a single rat, with recordings from 3 sessions. We took this conservative approach to control differences in nasal cavity structure that can vary across rats [60, 61], which may shape differences in ortho versus retro activity [11, 62]. See provided GitHub code for statistical summary of experimental data.

All procedures were carried out in accordance with the recommendations in the Guide for the Care and Use of Laboratory Animals of the National Institutes of Health and approved by University of Arkansas Institutional Animal Care and Use Committee (protocol #14049). Data were collected from 11 adult male rats (240–427 g; *Rattus Norvegicus*, Sprague-Dawley outbred, Harlan Laboratories, TX, USA) housed in an environment of controlled humidity (60%) and temperature (23°C) with 12h light-dark cycles. The experiments were performed in the light phase.

#### Surgical preparations

Anesthesia was induced with isoflurane inhalation and maintained with urethane (1.5 g/kg body weight (**bw**) dissolved in saline, intraperitoneal injection (**ip**)). Dexamethasone (2 mg/kg bw, ip) and atropine sulphate (0.4 mg/kg bw, ip) were administered before performing surgical procedures. Throughout surgery and electrophysiological recordings, core body temperature was maintained at 37°C with a thermostatically controlled heating pad. To isolate the effects of olfactory stimulation from breath-related effects, we performed a double tracheotomy surgery as described previously [11]. A Teflon tube (OD 2.1 mm, upper tracheotomy tube) was inserted 10mm into the nasopharynx through the rostral end of the tracheal cut. Another Teflon tube (OD 2.3 mm, lower tracheotomy tube) was inserted into the caudal end of the tracheal cut to allow breathing, with the breath bypassing the nasal cavity. Both tubes were fixed and sealed to the tissues using surgical thread. Local anesthetic (2% Lidocaine) was applied at all pressure points and incisions. Subsequently, a craniotomy was performed on the dorsal surface of the skull over the right olfactory bulb (2 mm × 2 mm, centered 8.5 mm rostral to bregma and 1.5 mm lateral from midline).

#### Olfactory stimulation

A Teflon tube was inserted into the right nostril and the left nostril was sealed by suturing. The upper tracheotomy tube inserted into the nasopharynx was used to deliver odor stimuli retronasally. Odorized air was delivered for 1 s in duration at 1 minute intervals, with a flow rate of 250 ml/min and 1% of saturated vapor. The odorant was Ethyl Butyrate (EB). We note that the full experimental data set included additional odors, but here we consider only EB.

#### Electrophysiology

A 32-channel microelectrode array (MEA, A4x2tet, NeuroNexus, MI, USA) was inserted 400 *μ*m deep from dorsal surface of OB targeting tufted and mitral cell populations. The MEA probe consisted of 4 shanks (diameter: 15 *μ*m, inter-shank spacing: 200 *μ*m), each with eight iridium recording sites arranged in two tetrode groups near the shank tip (inter-tetrode spacing: 150 *μ*m, within tetrode spacing 25 *μ*m). Simultaneous with the OB recordings, we recorded from a second MEA placed in anterior piriform cortex. Voltage was measured with respect to an AgCl ground pellet placed in the saline-soaked gel foams covering the exposed brain surface around the inserted MEAs. Voltages were digitized with 30 kHz sample rate (Cereplex + Cerebus, Blackrock Microsystems, UT, USA). Recordings were band-pass filtered between 300 and 3000Hz and semiautomatic spike sorting was performed using Klustakwik software, which is well suited to the type of electrode arrays used here [63].

## Supporting information

S1 Fig Statistically significant different spike count statistics. We performed two-sample t-tests assuming unequal variances for each point in time to assess whether the spike count statistics are significantly different with ortho and retro stimulation. We find statistical significance (*α* = 0.01) between ortho and retronasal firing rate (**A**) after and for the duration of odor stimulation (0.3 ≤ *t* ≤ 1 s with 100 ms time windows and 0.5 ≤ *t* ≤ 1.1 s with 200 ms time windows) as well as spike count covariance (**C**) for the entirety of the evoked state (0 ≤ *t* ≤ 2 s excluding *t* = 0 s with 200 ms time window). Spike count variance (**B**) is not found to have any statistical significant differences between ortho and retro. For completeness, significance of Fano Factor (**D**) and Pearson’s correlation (**E**) are also significantly different for ortho and retro in the evoked state (0 < *t* ≤ 2 s for Fano Factor and 0 ≤ *t* ≤ 2 s excluding *t* = 0 s with 100 ms time window for correlation).
(TIF)

S2 Fig Statistical measure of effect size using Cohen’s *d*. We calculated Cohen’s *d* value for the nondirectional (two-tailed) case to measure effect size index for t-tests of means (see S1 Fig) in standard units. We find small (*t* = 0.3, 0.7 ≤ *t* ≤ 0.9 s with 100 ms; 0.6 < *t* ≤1 s with 200 ms) and medium (0.3 < *t* < 0.7 s, *t* = 1 s with 100 ms; 0.4 ≤ *t* ≤0.6 s with 200 ms) effect size of statistical significance between ortho and retronasal firing rate (**A**) as well as small (0 ≤ *t* ≤ 2 s excluding *t* = 0 s with 200 ms time windows) effect size of spike count covariance (**C**). Spike count variance (**B**) does not have a measure of effect size since it is not found to have any statistical significant differences between ortho and retro. For completeness, effect size of Fano Factor (**D**) and Pearson’s correlation (**E**) are also found to be small (0 < *t* ≤0.2 s ∪ 0.4 < *t* ≤1 ∪ 1.4 < *t* ≤2 s for Fano Factor, and 0 ≤ *t* < 0.2 ∪ 0.5 < *t* < 0.8 excluding *t* = 0 s with 100 ms time windows for correlation) and medium (0.2 < *t* ≤0.4 ∪ 1 < *t* ≤1.4 s for Fano Factor and 0.2≤ *t* ≤ 0.5 ∪ 0.8 ≤ *t* ≤ 2 s for correlation).
(TIF)

S3 Fig Details of various ORN input rates we surveyed (achieved via trial and error) λ(*t*). **A)** Left: initial set of ORN inputs λ(*t*) (with evoked λ(*t*) = (*t* + 1)*e*^−(*t*+1)/*τ*^) we surveyed to better understand the MC firing rate (right), calculated with 2,000 realizations. **B)** Fitting the ortho firing rate well enough required considering many λ_*O*_(*t*), and we even shifted the spontaneous input rate up slightly at some point. However, the only 2 retro inputs we tried (pink and red) were relatively accurate.
(TIF)

S4 Fig ORN synapses statistic calculation is robust and accurate. Our theory for the ORN synaptic input statistics (Eqs (13), (18) and (22)) is accurate for time-varying inhomogeneous Poisson process rates and time-varying input correlation. **A)** Ortho-like input (fast rise and decay of Poisson rate) with same amplitude as retro (high), but with low input correlation used to capture data. Notice how the theory captures the fine structure of the covariance (double-hump). **B)** Retro-like input (slow rise and decay) with same amplitude as ortho (high), but with high input correlation used to capture data. **C, D)** Demonstrating accuracy of dynamic theory with much slower (unrealistic) time-scales: *τ*_1_ = 50 ms and *τ*_2_ = 100 ms and faster relative change in Poisson rate (all with low input correlation). Showing the quasi-steady-state approximation (Eqs (23)–(25)) in magenta. **C)** Sinusoidal input and time-varying amplitude: evoked λ(*t*) = 0.2 + 0.8(1 − 0.8 sin(−15*t*))(1 − *e*^−2*t*^), with synapse jump sizes *a*_1_ = 2, *a*_2_ = 5. **D)** Here the jump sizes have opposite signs to get negative covariances: *a*_1_ = 2, *a*_2_ = −1, with λ(*t*) = 2(*t* + 2.25)^2^ * (1 − 0.9 sin(10*t*))*e*^−|*t*−1|/0.35^. Gray curves (Monte Carlo) are much harder to see in **C,D** than in **A,B** because of the much larger magnitudes.
(TIF)

S5 Fig The LN fits to the OB model statistics are good overall. We consider 8 total different combinations of ORN inputs varying: temporal profile, amplitude height, input correlation (2 ways each). Despite the simplistic LN model, the resulting fits to the OB model are generally very good. The only exceptions are when the input correlation is relatively smaller, in which case the LN model does not accurately capture the evoked spike count covariance after several hundred milliseconds.
(TIF)
