# Supplementary figures and images for: Differences in olfactory bulb mitral cell spiking with ortho- and retronasal stimulation revealed by data-driven models
Source: PLoS Comput Biol. 2021 Sep 20;17(9):e1009169. doi: 10.1371/journal.pcbi.1009169 (PMC8483419; doi:10.1371/journal.pcbi.1009169)

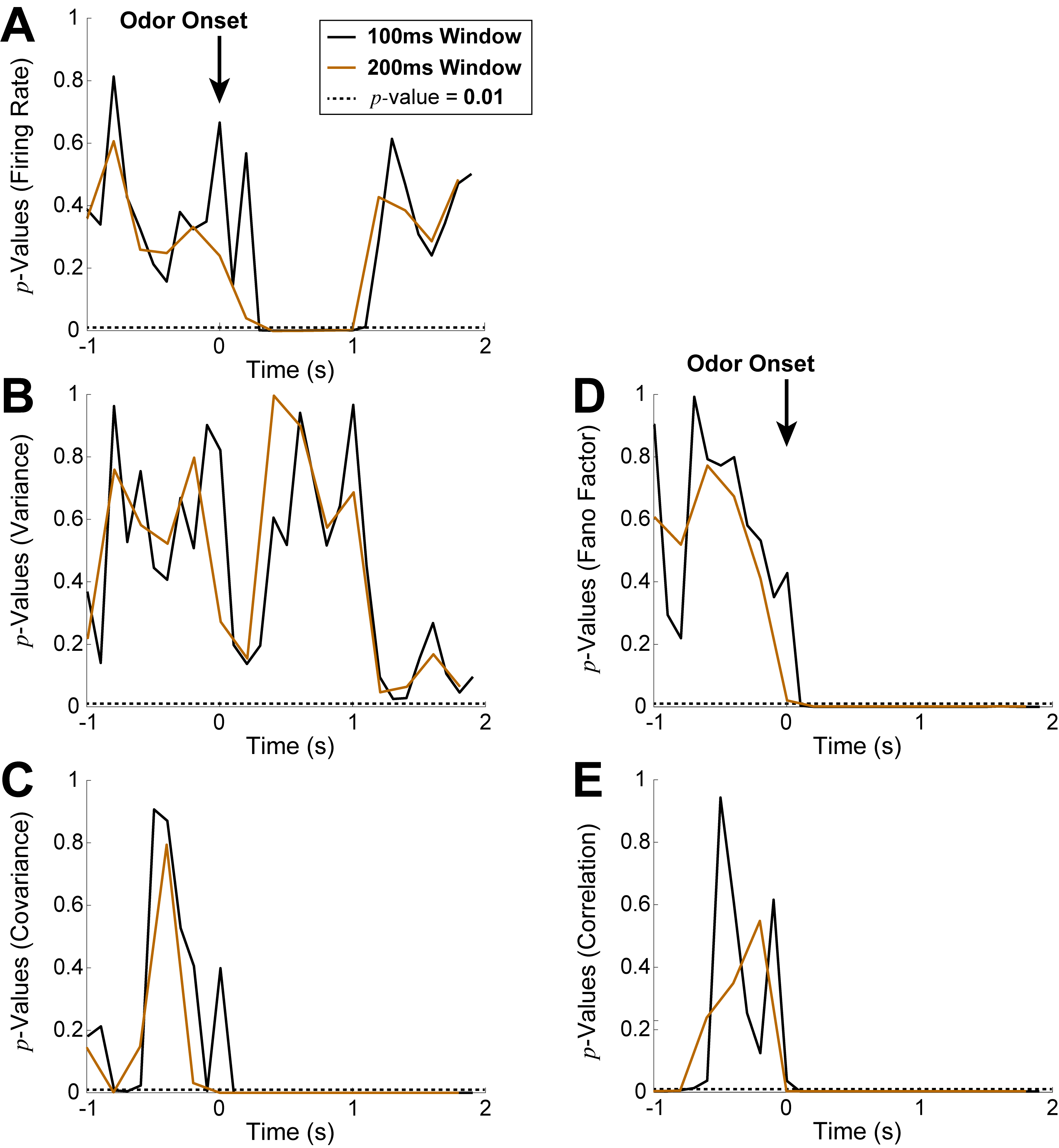

Supplement: S1 Fig — We performed two-sample t-tests assuming unequal variances for each point in time to assess whether the spike count statistics are significantly different with ortho and retro stimulation. We find statistical significance (α = 0.01) between ortho and retronasal firing rate (A) after and for the duration of odor stimulation (0.3 ≤ t ≤ 1 s with 100 ms time windows and 0.5 ≤ t ≤ 1.1 s with 200 ms time windows) as well as spike count covariance (C) for the entirety of the evoked state (0 ≤ t ≤ 2 s excluding t = 0 s with 200 ms time window). Spike count variance (B) is not found to have any statistical significant differences between ortho and retro. For completeness, significance of Fano Factor (D) and Pearson’s correlation (E) are also significantly different for ortho and retro in the evoked state (0 < t ≤ 2 s for Fano Factor and 0 ≤ t ≤ 2 s excluding t = 0 s with 100 ms time window for correlation). (TIF) [file pcbi.1009169.s001.tif]

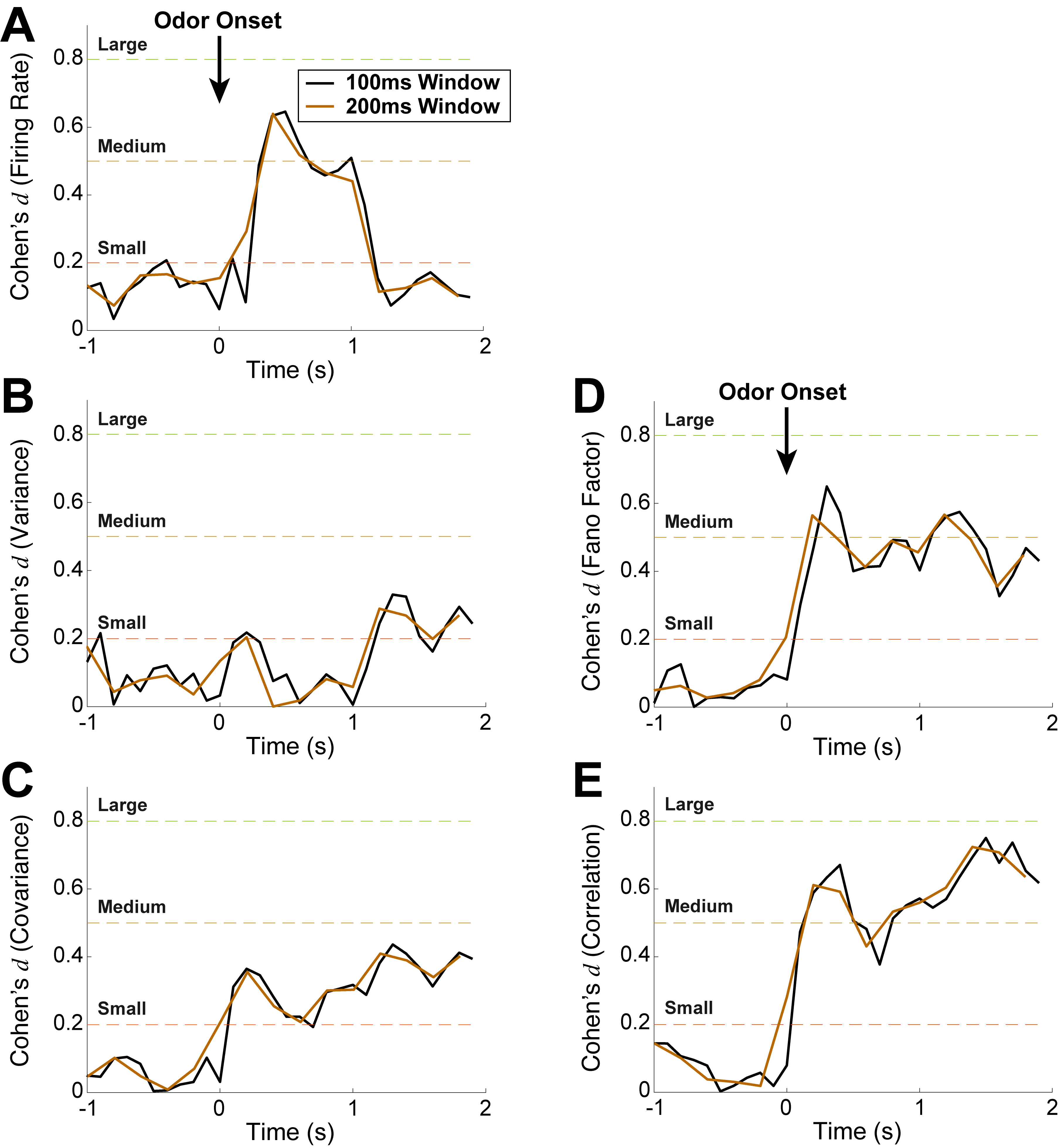

Supplement: S2 Fig — We calculated Cohen’s d value for the nondirectional (two-tailed) case to measure effect size index for t-tests of means (see S1 Fig) in standard units. We find small (t = 0.3, 0.7 ≤ t ≤ 0.9 s with 100 ms; 0.6 < t ≤1 s with 200 ms) and medium (0.3 < t < 0.7 s, t = 1 s with 100 ms; 0.4 ≤ t ≤0.6 s with 200 ms) effect size of statistical significance between ortho and retronasal firing rate (A) as well as small (0 ≤ t ≤ 2 s excluding t = 0 s with 200 ms time windows) effect size of spike count covariance (C). Spike count variance (B) does not have a measure of effect size since it is not found to have any statistical significant differences between ortho and retro. For completeness, effect size of Fano Factor (D) and Pearson’s correlation (E) are also found to be small (0 < t ≤0.2 s ∪ 0.4 < t ≤1 ∪ 1.4 < t ≤2 s for Fano Factor, and 0 ≤ t < 0.2 ∪ 0.5 < t < 0.8 excluding t = 0 s with 100 ms time windows for correlation) and medium (0.2 < t ≤0.4 ∪ 1 < t ≤1.4 s for Fano Factor and 0.2≤ t ≤ 0.5 ∪ 0.8 ≤ t ≤ 2 s for correlation). (TIF) [file pcbi.1009169.s002.tif]

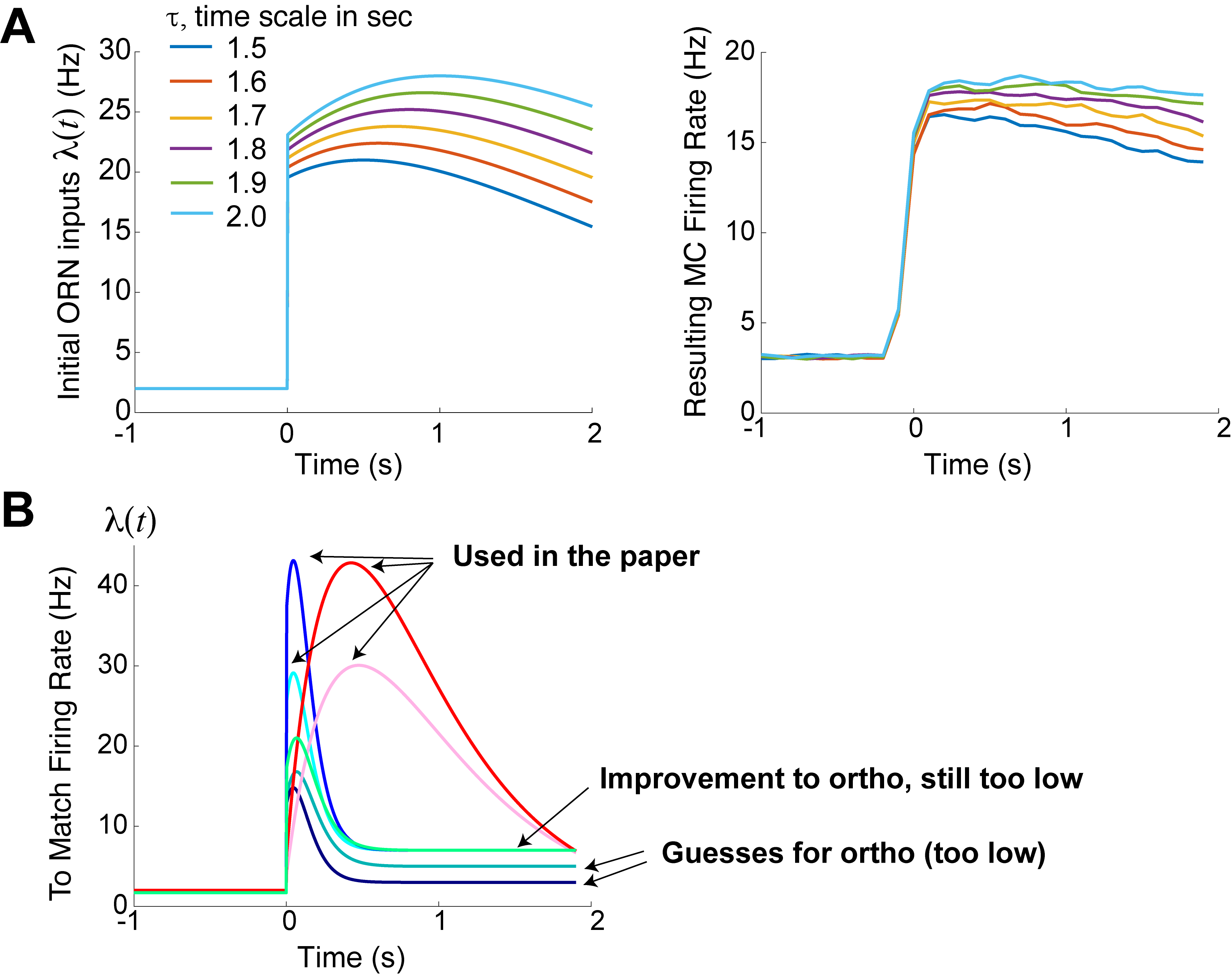

Supplement: S3 Fig — A) Left: initial set of ORN inputs λ(t) (with evoked λ(t) = (t + 1)e−(t+1)/τ) we surveyed to better understand the MC firing rate (right), calculated with 2,000 realizations. B) Fitting the ortho firing rate well enough required considering many λO(t), and we even shifted the spontaneous input rate up slightly at some point. However, the only 2 retro inputs we tried (pink and red) were relatively accurate. (TIF) [file pcbi.1009169.s003.tif]

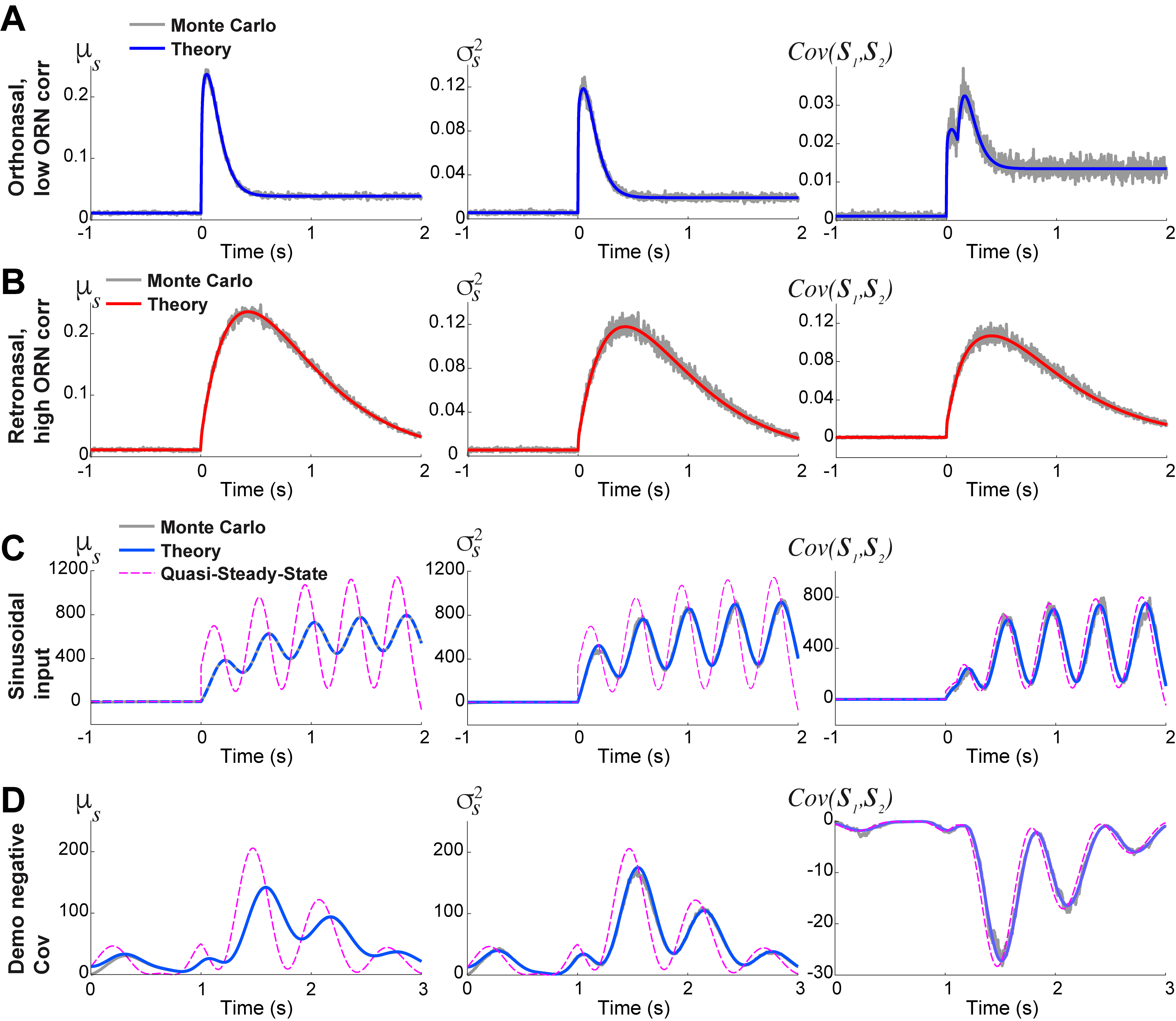

Supplement: S4 Fig — Our theory for the ORN synaptic input statistics (Eqs (13), (18) and (22)) is accurate for time-varying inhomogeneous Poisson process rates and time-varying input correlation. A) Ortho-like input (fast rise and decay of Poisson rate) with same amplitude as retro (high), but with low input correlation used to capture data. Notice how the theory captures the fine structure of the covariance (double-hump). B) Retro-like input (slow rise and decay) with same amplitude as ortho (high), but with high input correlation used to capture data. C, D) Demonstrating accuracy of dynamic theory with much slower (unrealistic) time-scales: τ1 = 50 ms and τ2 = 100 ms and faster relative change in Poisson rate (all with low input correlation). Showing the quasi-steady-state approximation (Eqs (23)–(25)) in magenta. C) Sinusoidal input and time-varying amplitude: evoked λ(t) = 0.2 + 0.8(1 − 0.8 sin(−15t))(1 − e−2t), with synapse jump sizes a1 = 2, a2 = 5. D) Here the jump sizes have opposite signs to get negative covariances: a1 = 2, a2 = −1, with λ(t) = 2(t + 2.25)2 * (1 − 0.9 sin(10t))e−|t−1|/0.35. Gray curves (Monte Carlo) are much harder to see in C,D than in A,B because of the much larger magnitudes. (TIF) [file pcbi.1009169.s004.tif]

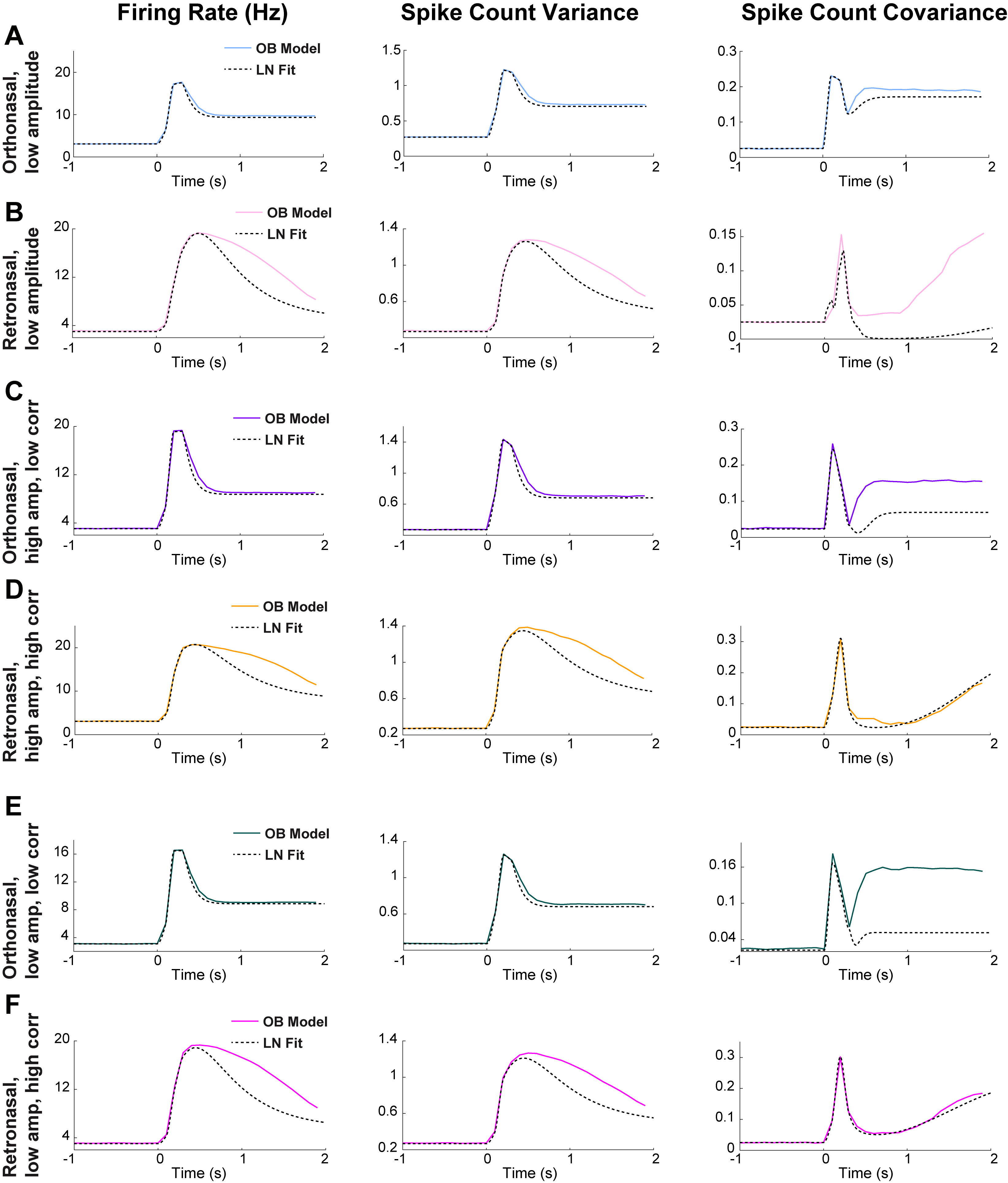

Supplement: S5 Fig — We consider 8 total different combinations of ORN inputs varying: temporal profile, amplitude height, input correlation (2 ways each). Despite the simplistic LN model, the resulting fits to the OB model are generally very good. The only exceptions are when the input correlation is relatively smaller, in which case the LN model does not accurately capture the evoked spike count covariance after several hundred milliseconds. (TIF) [file pcbi.1009169.s005.tif]
